# Transformation of doped graphite into cluster-encapsulated fullerene cages

**DOI:** 10.1038/s41467-017-01295-9

**Published:** 2017-10-31

**Authors:** Marc Mulet-Gas, Laura Abella, Maira R. Cerón, Edison Castro, Alan G. Marshall, Antonio Rodríguez-Fortea, Luis Echegoyen, Josep M. Poblet, Paul W. Dunk

**Affiliations:** 1 National High Magnetic Field Laboratory, Florida State University, Tallahassee, FL 32310 USA; 2 Departament de Química Física i Inorgànica, Universitat Rovira i Virgili, Tarragona, 43007 Spain; 3 Department of Chemistry, University of Texas at El Paso, El Paso, TX 79968 USA; 4 Department of Chemistry and Biochemistry, Florida State University, Tallahassee, FL 32306 USA

## Abstract

An ultimate goal in carbon nanoscience is to decipher formation mechanisms of highly ordered systems. Here, we disclose chemical processes that result in formation of high-symmetry clusterfullerenes, which attract interest for use in applications that span biomedicine to molecular electronics. The conversion of doped graphite into a C_80_ cage is shown to occur through bottom-up self-assembly reactions. Unlike conventional forms of fullerene, the iconic Buckminsterfullerene cage, *I*_h_-C_60_, is entirely avoided in the bottom-up formation mechanism to afford synthesis of group 3-based metallic nitride clusterfullerenes. The effects of structural motifs and cluster–cage interactions on formation of compounds in the solvent-extractable C_70_–C_100_ region are determined by in situ studies of defined clusterfullerenes under typical synthetic conditions. This work establishes the molecular origin and mechanism that underlie formation of unique carbon cage materials, which may be used as a benchmark to guide future nanocarbon explorations.

## Abstract (web summary)

An understanding of how caged carbon materials self-assemble from doped graphite is a long-standing challenge. Here, the authors show that distinct bottom-up processes lead to the synthesis of high-symmetry clusterfullerenes.

## Introduction

Fullerenes that encapsulate clusters of atoms represent a fundamental interest in chemistry, materials, and carbon science due to their unique properties and nanoscale structures^1–3^. Compounds that entrap the trimetallic nitride cluster are among the most intensively studied form of molecular nanocarbon because they offer promise as contrast agents and other biomedical diagnostics, photovoltaics, and molecular electronics^4–7^. In particular, Sc_3_N@*I*_h_-C_80_ mysteriously forms as the “third most abundant fullerene”, only empty C_60_ and C_70_ have been isolated in higher yield^8, 9^. The M_3_N (M = metal) cluster imparts stability to cage sizes from ~C_70_ to C_100_ and donates six electrons to the carbon cage^10, 11^. Nitride clusterfullerene (NCF) compounds possess diverse structural motifs that are relevant to other carbon networks, such as nanotubes and graphene. For example, non-isolated pentagon rule (non-IPR), Sc_3_N@*D*_3_-C_68_, as well as an isomer of the C_66_ cage exhibit multiple configurations of fused pentagons^12–14^. Very recently, a heptagon-containing structure was characterized as the NCF, Sc_2_LaN@C_80_(hept)^15^.

An understanding of how these compounds form by simple vaporization of doped graphite is paramount because the intrinsic mechanisms and chemical principles that control formation may be exploited to create entirely new forms of nanomaterials and overcome obstructions in synthesis of cluster-encapsulated carbon materials. Recently, top-down proposals have been rationally inferred as a possible route to formation of the archetypal NCF, M_3_N@*I*_h_-C_80_, and other high-symmetry fullerenes based on molecular evidence, computational studies, and observations of graphene under electron beam irradiation^16–19^. In this case, carbon sheets are envisioned to warp into giant cages and subsequently shrink into the icosahedral C_80_ cage. However, it remains unknown how an icosahedral cage that entraps a four-atom cluster may self-assemble from graphite vaporization, because in situ studies are not possible by conventional synthesis methods. Such in situ studies are a challenging endeavor, but are crucial by virtue that carbon is an extraordinary element that possesses the tendency to exhibit different chemistry under conditions that diverge from characteristic synthesis due to its versatile bonding properties. Interestingly, the conversion of polycyclic aromatic hydrocarbons or their derivatives to fullerenes shares some conceptual similarity to those proposed top-down mechanisms^20^.

Here, we show that clusterfullerenes are formed from doped graphite, a universal starting material for carbon nanostructure synthesis, by a laser-based synthesis method that permits in situ formation investigations and uncover unprecedented mechanistic insight into self-assembly of complex carbon compounds. We disclose that the formation of high-symmetry clusterfullerenes occurs through a distinct bottom-up mechanism and that high-yield formation of Sc_3_N@C_80_ is achieved by the complete circumvention of Buckminsterfullerene, C_60_^21^, in bottom-up reaction paths. To validate our model and probe cage selection and cluster size effects, we directly study the small, non-IPR compound, Sc_3_N@*D*_3_-C_68_, and larger, high-symmetry species, including M_3_N@*I*_h_-C_80_ (M = group 3 metal), under precise synthetic conditions.

## Results

### M_3_N-encapsulated (M = group 3) cages from doped graphite

**Fig. 1:**
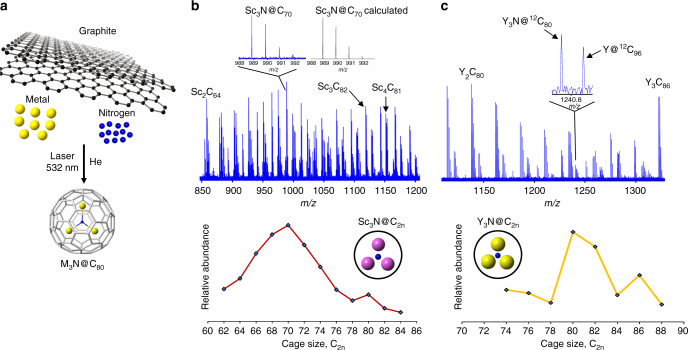
Clusterfullerenes formed by laser vaporization of group 3 metal-doped and nitrogen-doped graphite. **a** Synthesis schematic for clusterfullerenes formed from a mixture of graphite, metal oxide, and melamine (nitrogen source) in this work. FT-ICR mass spectra of cluster cations generated by laser vaporization of **b** Sc-doped and N-doped graphite and **c** Y-doped and N-doped graphite. M_3_N@C_2n_ formation distributions are graphically shown below each spectrum

To devise a strategy to study in situ self-assembly processes for these nanomaterials, we performed extensive analyses by laser vaporization of graphite-based starting materials doped with group 3 metal oxides and numerous sources of heteroatoms, such as gaseous (e.g., ammonia and N_2_) and molecular nitrogen sources. In fact, these heteroatom sources are used in arc discharge synthesis to produce macroscopic quantities of the compounds^22^. In our approach, online chemical sampling is carried out by use of a pulsed laser vaporization cluster source, analyzed by state-of-the-art Fourier transform ion cyclotron resonance (FT-ICR) mass spectrometry, which was previously limited to empty cages and conventional metallofullerenes^23–25^. We find that the solid organic nitrogen sources^26^ are an excellent choice for the formation of trimetallic NCFs under the present laser-based conditions and, in particular, melamine yields highly reproducible formation products^27, 28^. In these strongly ionizing environments, positive ions are expected to be representative of the neutral NCF distributions, similar to empty cages and mono-metallofullerenes^23^. Figure 1 shows molecular nanocarbon products formed by laser vaporization (532 nm, 10 mJ per pulse) of a stationary target rod comprised of graphite, scandium oxide, and melamine (10% atom Sc, 1:2 ratio for Sc:C_3_H_6_N_6_) in a He atmosphere. Surprisingly, the small compounds, such as Sc_3_N@C_68_, Sc_3_N@C_70_, Sc_3_N@C_72_, and Sc_3_N@C_74_, exhibit higher relative abundance than Sc_3_N@C_80_ and similar sized cages. That Sc_3_N@C_80_ nanocluster, however, displays an enhanced abundance compared to other medium-sized cages and is a “magic numbered” species. Under the present clusterfullerene-generating conditions, empty cage fullerenes are suppressed by laser synthesis, similar to observations for arc discharge synthesis from doped graphite containing these particular starting materials. Notably, all Sc_3_N@C_2n_ (C_2n_ = 68, 70, 78, 80, 82) synthesized and isolated by means of the arc discharge methods correspond to the observed cage sizes^2, 29^.

Although the smallest species, Sc_3_N@C_62_-C_66_, are formed in abundance under the present conditions, they may “react away” in the solid state or upon exposure to air or solvent. Therefore, some of these pristine isomers may not be readily detectable in arc discharge extracts or soot, as for other smaller non-IPR fullerenes^30^. Another possibility is that more carbon vapor may be available for insertion reactions in arc discharge than for the present laser vaporization conditions. However, it is clear that the Sc_3_N@C_80_ clusterfullerene is an abundantly produced medium-sized Sc_3_N@C_2n_ formed by laser vaporization of doped graphite. At lower laser fluence (~5 mJ), we find that the smallest clusterfullerene cages, Sc_3_N@C_2n_ (C_2n_ = C_62_ to ~C_74_), are formed but medium-sized species, such as Sc_3_N@C_80_, are not observed. These results show that the smallest Sc_3_N-based clusterfullerenes appear to form before the larger compounds from doped graphite under our conditions.

Structural analysis of Sc_3_N@C_2n_ produced from the bulk graphite-based starting material is obtained by collision-induced dissociation (CID) experiments, performed by means of sustained off-resonance irradiation (SORI)^31^. Supplementary Fig. 1 identifies the fragmentation pattern of gas-phase isolated Sc_3_N@C_70_ formed by laser vaporization of the graphite/Sc_2_O_3_/melamine mixture. The singular dissociation pathway observed for Sc_3_N@C_70_ in an ultrahigh vacuum is a C_2_-elimination event. The internally bound cluster, Sc_3_N, remains trapped within the nanoscale void of the carbon cage when highly thermally excited by collisions, whereas any exohedrally bound metals or heteroatoms would readily dissociate. Therefore, dissociation investigations provide compelling evidence that Sc_3_N@C_70_ exhibits a NCF structure. Larger Sc_3_N@C_2n_ formation products display that same characteristic clusterfullerene dissociation pattern.

To discern the influence of the M_3_N cluster size effect with respect to the step of initial cage nucleation by means of bottom-up formation from starting material plasma, Y-doped melamine-containing graphite is examined under identical conditions. The larger ionic radius of Y (0.90 Å), and thus larger M_3_N cluster, compared to Sc (0.75 Å), provides a mechanistic avenue to experimentally probe that process. A striking change in formation distribution is observed for Y_3_N@C_2n_ compared to Sc_3_N@C_2n_ (Fig. 1). The small M_3_N-based clusterfullerenes observed for Sc_3_N@C_2n_ are entirely absent, and, instead, nanocarbons are shifted to cage sizes of Y_3_N@C_74_ to Y_3_N@C_88_^32^. However, the Y_3_N@C_80_ molecular ion, like Sc_3_N@C_80_, exhibits higher relative abundance indicating formation of a stable C_80_ isomer. The Y_3_N@C_2n_ compounds, for example, Y_3_N@C_80_, are confirmed to be endohedral NCFs by SORI-CID investigations, as expected. Other families of metallofullerenes are detected from both Sc-doped and Y-doped carbon plasma systems and correspond to M_2_C_2n_, M_3_C_2n_, M_4_C_2n_, as well as odd carbon-numbered clusters such as M_3_C_n_ that must contain at least one C within a cage^33^. Thus, in addition to the successful formation of NCFs, numerous forms of clusterfullerenes are also produced from doped graphite by laser vaporization. Importantly, the observed cage size shift for Sc_3_N compared to Y_3_N clarifies that the M_3_N cluster nucleates formation of the smallest cages in the first bottom-up step.

Sc_3_N@C_2n_ are formed in higher relative abundance than Y_3_N@C_2n_ generated by laser vaporization of the starting materials, and therefore Sc_3_N is more efficiently entrapped within cages under our conditions. Thus, Sc appears to possess a special ability to bond with C and/or N in the initial nucleation step. Moreover, molecular ions that contain up to four Sc atoms are readily observed from Sc/N/C condensing plasma, whereas at most three Y atoms can be encapsulated from the Y/N/C plasma under the same vaporization parameters. These results provide mechanistic insight into how Sc may “pull in” other elements into cages through initial formation of the smallest NCFs^34^, which we propose grow into larger species through bottom-up reactions as we clearly demonstrate below.

### Transformation of Sc_3_N@*D*_3_-C_68_ into Sc_3_N@C_80_

**Fig. 2:**
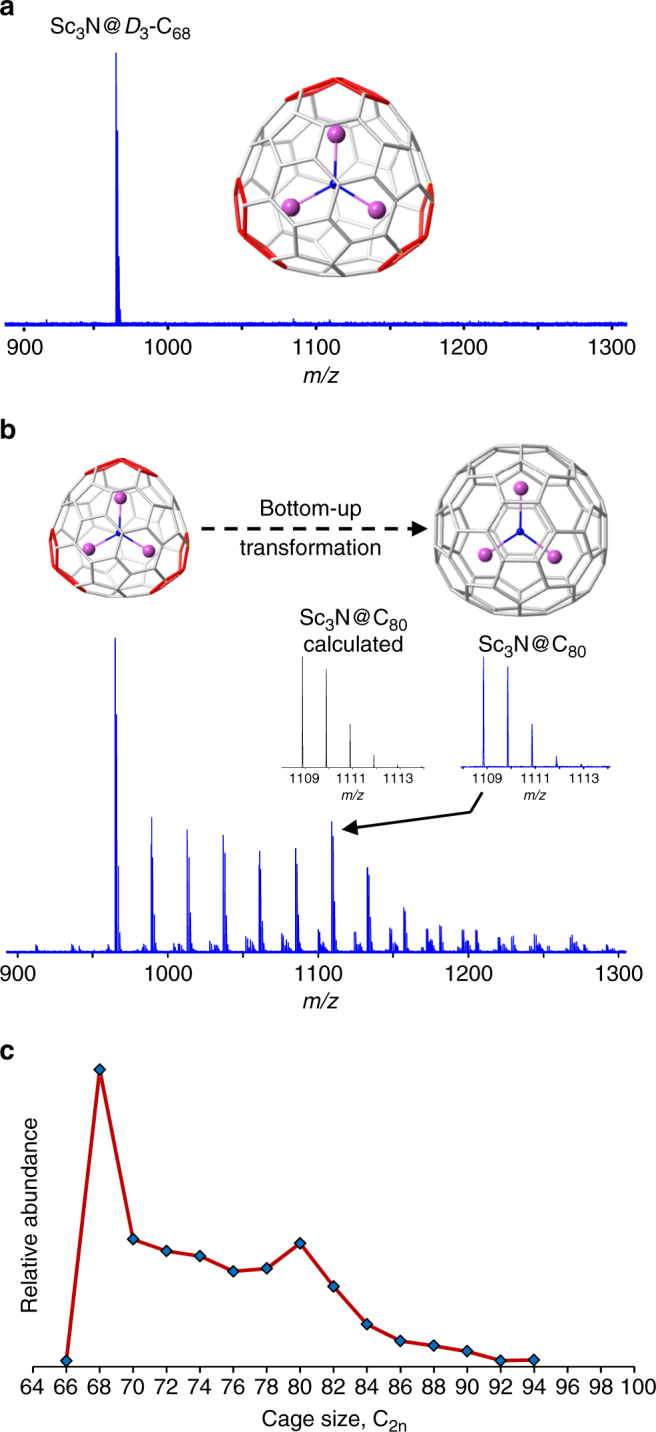
Bottom-up growth of a small, fused pentagon-containing clusterfullerene. **a** Low energy (~2 mJ) laser desorption spectrum (positive ions) of isomerically pure, Sc_3_N@*D*_3_-C_68_, without exposure to carbon vapor from graphite. **b** Molecular reactivity and behavior of Sc_3_N@C_68_ in carbon vapor from graphite in a He atmosphere (~10 mJ per pulse). **c** Growth distribution for Sc_3_N@C_68_ + *n*C_2_

Chemical processes involved in the growth of the initially formed, small Sc_3_N-based clusterfullerenes from bulk starting materials are elucidated by analysis of isomerically pure NCF cages in carbon vapor generated from graphite, conducted under the same high energy formation conditions (10 mJ per pulse). These studies also provide insight into structural effects with respect to nanocarbon reactions that operate during self-assembly. Figure 2 shows that Sc_3_N@*D*_3_-C_68_ unambiguously exhibits bottom-up growth by carbon insertion reactions that results in formation of larger Sc_3_N@C_2n_ (C_2n_ = 70–94) compounds. Under the present conditions, the most abundant growth product is Sc_3_N@C_80_, which exhibits an enhanced abundance compared to other medium-sized cages, similar to Sc_3_N@C_80_ generated by laser vaporization of bulk doped graphite (Fig. 1). Thus, in both cases, the results indicate that a stable, high-symmetry C_80_ cage isomer is formed. Dissociation investigations confirm that Sc_3_N is encapsulated in clusterfullerene growth product cages (Supplementary Figs. 2, 3).

To distinguish formation mechanisms for the small Sc_3_N@C_2n_ (C_2n_ = C_62_–C_66_) products synthesized by laser vaporization of Sc-doped and N-doped graphite, which have not been detected in extracts or soot from arc discharge, Sc_3_N@*D*_3_-C_68_ is studied in the absence of graphite vapor under the high energy conditions of synthesis. Supplementary Fig. 4 shows products generated from Sc_3_N@*D*_3_-C_68_ by direct laser ablation (10 mJ per pulse) without exposure to carbon vapor. Surprisingly, Sc_3_N@C_70_, a bottom-up growth structure, is the most abundant molecular reaction product, suggesting that carbon insertion reactions can be favored in low carbon density, high-energy conditions. Sc_2_N@C_68_, a Sc-loss product, and Sc_3_N@C_66_, formed by C_2_-loss, are observed only in very low abundance. Consequently, these results provide additional evidence that the smallest Sc_3_N@C_2n_ (C_2n_ = C_62_–C_66_) are not formed from larger Sc_3_N@C_2n_ by top-down processes during self-assembly from graphite starting material and are consistent with a bottom-up formation mechanism.

Reaction products that correspond to non-NCF compounds, namely, Sc_3_NC_n_, Sc_2_NC_n_, and ScNC_n_ (C_n_ = odd number of C atoms) also result after growth of Sc_3_N@*D*_3_-C_68_ in carbon vapor (Supplementary Fig. 5). We find that all odd-numbered carbon chemical compositions dissociate by C_2_-elimination with retention of an odd number of carbon and all Sc and N atoms. Consequently, the presence of a carbon adatom is excluded because it readily dissociates at low thermal energy. Therefore, all of these molecular products contain clusters of Sc, N, and C within cages. As a result of the present high energy formation conditions that render compounds with no C adatoms, the Sc_3_NC_n_ species plausibly exhibit structures, Sc_3_NC@C_2n_, in which the five-atom cluster, Sc_3_NC, is entrapped within even-numbered carbon cages. These species result from intramolecular reactions that take place during the growth process. Notably, the smallest member of Sc_3_NC_n_ observed from Sc_3_N@*D*_3_-C_68_ growth is Sc_3_NC_81_ (Supplementary Fig. 5), which may be a contributing formation route to Sc_3_NC_81_ that has been isolated by means of arc discharge and exhibits a carbonitride clusterfullerene structure, Sc_3_NC@C_80_^35^.

### Computational analysis for reaction paths from C_68_ to C_80_

**Fig. 3:**
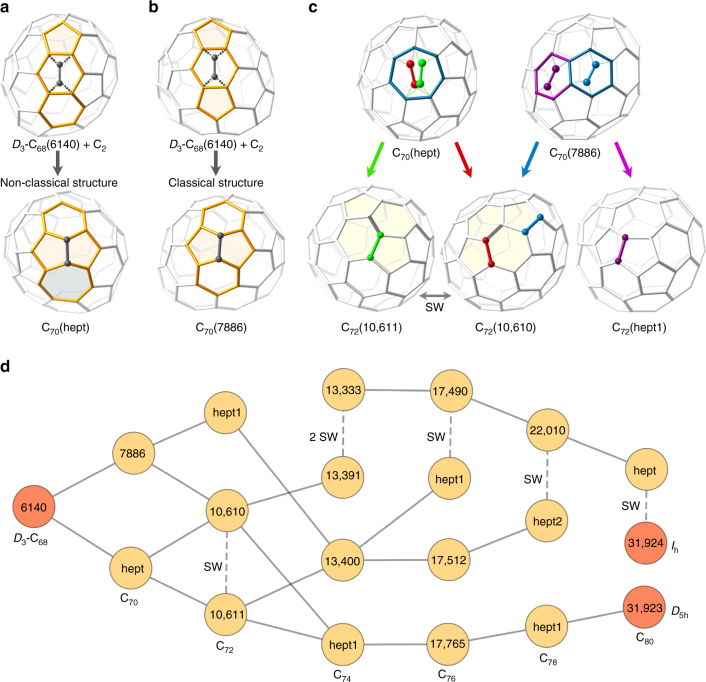
Reaction paths to high-symmetry C_80_ isomers (*I*_h_, *D*_5h_) from *D*_3_-C_68_ through non-classical and classical cages. *D*_3_-C_68_ can grow by C_2_ insertion into the **a** non-classical structure that contains a heptagon motif, C_70_(hept), or **b** a classical structure comprised of only pentagons and hexagons, C_70_(7886). **c** For the next bottom-up cage transformation, C_70_ to C_72_, the most plausible isomers are found to be the classical cages, C_72_(10611), C_72_(10610), and heptagon-containing C_72_(hept1). **d** From those C_72_ isomers, reaction paths to high-symmetry C_80_ cages, *I*_h_-C_80_ and *D*_5h_-C_80_, involve C_2_ insertion reactions and two to three SW rearrangements through classical and non-classical cage intermediates

Sc_3_N@*D*_3_-C_68_ is clearly demonstrated to transform into Sc_3_N@C_70_ after exposure to carbon vapor generated from graphite, which involves the overall incorporation of C_2_ into the caged network. That nanocluster is also among the most abundant NCFs formed by laser vaporization of Sc-doped and N-doped graphite in this work. Interestingly, the only Sc_3_N@C_70_ species that has been isolated is C_70_(7854)^36^. Note that we will now use the spiral algorithm numerical identifier in parenthesis to distinguish isomers for a particular cage size^37^. To discern a reaction path for the Sc_3_N@*D*_3_-C_68_ to Sc_3_N@C_70_ transformation, all possible topological C_2_ insertions were analyzed for the *D*_3_-C_68_(6140) cage in the hexaanionic form, C_2n_^6−^, to account for charge transfer from the encapsulated Sc_3_N cluster to the cage. Six isomers of C_70_ can be generated by direct C_2_ insertion (Supplementary Fig. 6) without the involvement of Stone-Wales (SW) bond rearrangements. The two lowest energy product structures, shown in Fig. 3a, b, are found to be associated with very exothermic energies (Supplementary Table 1). C_70_(7886) exhibits a classical structure comprised of pentagons and hexagons, whereas C_70_(hept) possesses a non-classical structure that contains a heptagon motif.

Analysis of all possible C_2_ insertions into these predicted C_70_ isomers (Supplementary Fig. 7) is performed for the next bottom-up cage transformation and plausible C_72_ intermediates are found to be C_72_(10611), C_72_(10610), and C_72_(hept1), as shown in Fig. 3c. Notably, C_72_(10611) acts as a “gateway” cage and is intimately related by a single SW rearrangement (Supplementary Fig. 8) to C_72_(10610) with a barrier (~150 kcal mol^−1^) that can be surpassed at the temperature of fullerene formation (>1000 K). Although the heptagon structure is 24.2 kcal mol^−1^ higher in energy than C_72_(106011), we find that the energy profiles for these cage growth transformations are similar (Supplementary Fig. 9). Therefore, formation of classical and heptagon-containing cages should take place during bottom-up clusterfullerene growth, which is in agreement with the recent characterization of trimetallic nitride, LaSc_2_N@C_80_(hept), as well as for larger heptagon-containing cage sizes^15, 38, 39^. Structures that possess two heptagons are not considered because such cages are very strained and are at least 54 kcal mol^−1^ higher in energy than C_72_(10611). Reaction energies for all of the lowest energy isomers were computed in the clusterfullerene form for comparison to the hexaanionic investigations. Supplementary Table 2 shows that reaction energies are somewhat lower, but remain very exothermic. Thus, formation of the proposed C_70_ and C_72_ growth structures is favorable based on thermodynamic considerations. A detailed description of the growth mechanism is given in Supplementary Fig. 9.

We have investigated global pathways to high-symmetry C_80_ clusterfullerenes from Sc_3_N@*D*_3_-C_68_ based on this strategy for step-by-step cage formations of low energy isomers through C_2_ insertions and bond rearrangements. Despite the complexity of the processes involved (Supplementary Fig. 10), it is extraordinary that several relatively simple pathways exist and are shown in Fig. 3d. Initiating from the proposed C_68_ to C_72_ structure progression, we find reaction paths from *D*_3_-C_68_ to icosahedral C_80_ that involve the known NCF cages, C_78_(22010), C_80_(hept), and C_80_(*I*_h_) in final reaction sequence, where C_80_(hept) is the structure of recently isolated NCF, LaSc_2_@C_80_(hept)^15, 40–42^. Strikingly, Fig. 3d also shows that a bottom-up sequence of six direct C_2_ insertions without any SW rearrangement results in the known high-symmetry *D*_5h_-C_80_ cage, M_3_N@*D*_5h_-C_80_(31923). Thus, we find that all plausible bottom-up growth routes to high-symmetry C_80_ involve a total of six C_2_ insertion reactions and two to three C_2_ rearrangements. Importantly, reaction energies, and free energies computed at the high temperature of synthesis (2000 K), for all routes are confirmed to be very exothermic (Supplementary Tables 2–4). It is possible that some of these predicted non-classical and classical intermediates may be isolated and characterized in the near future (Fig. 3, Supplementary Fig. 10, and Supplementary Table 5).

### Growth of M_3_N@C_80_ into larger, lower symmetry cages

**Fig. 4:**
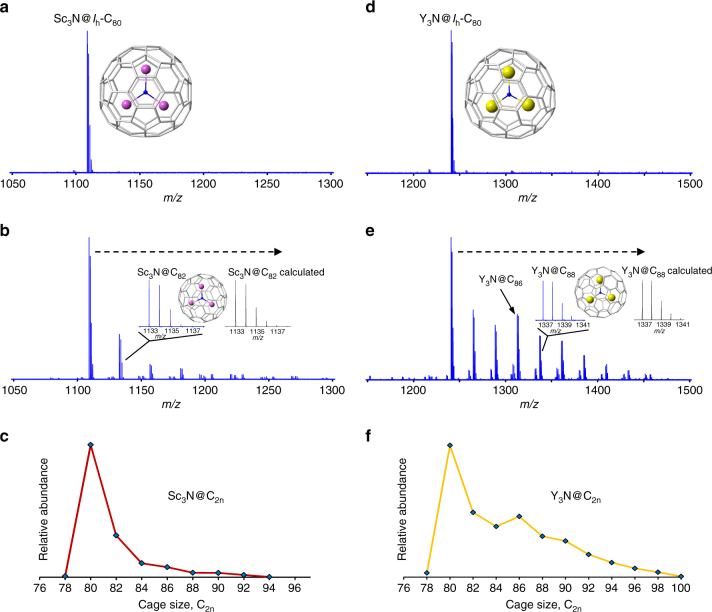
Influence of the encapsulated cluster on growth of icosahedral C_80_. Isomerically pure **a** Sc_3_N@*I*_h_-C_80_ after laser desorption (~2 mJ) without carbon vapor and **b** after reaction with graphite vapor in He (10 mJ) and **c** Sc_3_N@C_2n_ + *n*C_2_ formation distribution. Comparison of isomerically pure **d** Y_3_N@*I*_h_-C_80_ after laser desorption (~2 mJ) and **e** reaction with graphite vapor under identical conditions (10 mJ) and **f** Y_3_N@C_2n_ + *n*C_2_ formation distribution. C_2_-elimination events are not readily observed for either M_3_N@*I*_h_-C_80_

As a crucial test for our proposed bottom-up formation mechanism from graphite, the M_3_N@*I*_h_-C_80_ compound is specifically investigated under the harsh conditions typical of plasma synthesis, thereby providing important nanocarbon mechanistic information because of its icosahedral symmetry and medium cage size. Figure 4a–c shows the products formed after exposure of isomerically pure Sc_3_N@*I*_h_-C_80_ to carbon plasma in He. An identical laser fluence (10 mJ per pulse) is used for all molecular reactivity studies in this work to facilitate comparison to gas-phase synthesis of Sc_3_N@C_2n_ from raw starting materials without pre-existing NCFs. Numerous larger metallic nitride species are formed by repeated carbon insertion reactions into the Sc_3_N@*I*_h_-C_80_ precursor. The dominant nanocarbon reaction after exposure of Sc_3_N@*I*_h_-C_80_ to graphite vapor is a single C_2_ insertion to yield Sc_3_N@C_82_. By contrast, species smaller than Sc_3_N@C_80_ are absent, including Sc_3_N@C_68_, revealing that carbon loss or top-down formation to smaller cages is not a significant process under the high energy, high carbon density conditions of synthesis. The structures of the molecular product ions Sc_3_N@C_2n_ (C_2n_ ≥ 82) formed through bottom-up growth of Sc_3_N@*I*_h_-C_80_ are confirmed (Supplementary Fig. 11) to be trimetallic NCFs. In addition, we find that carbon loss reactions do not readily take place when Sc_3_N@*I*_h_-C_80_ is subjected to high energy laser ablation (10 mJ per pulse) without carbon vapor, i.e., high energy, low carbon density conditions (Supplementary Fig. 12), a molecular behavior consistent with the much smaller Sc_3_N@*D*_3_-C_68_. The *D*_5h_-C_80_ isomer encapsulated by Sc_3_N was also probed in graphite vapor and exhibits bottom-up growth behavior, and it appears to be slightly more reactive than *I*_h_-C_80_ (Supplementary Fig. 13).

To probe cluster-cage size effects with respect to carbon insertion reactions for the *I*_h_-C_80_ cage, molecular reactivity studies are performed whereby Y_3_N is substituted for Sc_3_N. Figure 4d–f shows products that result after exposure of isomerically pure Y_3_N@*I*_h_-C_80_ to graphite vapor, conducted under identical conditions to those for the Sc_3_N@*I*_h_-C_80_ (and Sc_3_N@*D*_3_-C_68_) growth experiments. Y_3_N@C_82_, formed by C_2_ incorporation, is formed in high relative abundance; however, Y_3_N@C_84_ and Y_3_N@C_2n_ compounds as large as Y_3_N@C_100_ are present in substantial abundance. In addition, Y_3_N@*I*_h_-C_80_, like Sc_3_N@*I*_h_-C_80_, does not readily exhibit carbon loss events or top-down behavior (Supplementary Fig. 14) when subjected to high energy laser ablation in absence of carbon vapor (i.e., low carbon density, high energy conditions). Extensive SORI-CID experiments strongly support that the major growth products are homogeneous Y-based NCFs (Supplementary Fig. 15). The more extensive bottom-up growth of Y_3_N@*I*_h_-C_80_ is in agreement with the formation distribution of Y_3_N@C_2n_ generated from Y-doped and N-doped graphite (Fig. 1). Thus, the bottom-up mechanism is attributed to also operate in the Y-containing graphite starting material plasma.

Further evidence to corroborate metallic NCF structures for Y_3_N@C_2n_ growth products, as well as Sc_3_N@C_2n_, synthesized in this work is obtained by dissociation analysis of the endohedral heterofullerene, Y_2_@C_79_N, which exhibits an 80 atom cage that substitutes N for a single C atom in the caged network^43^. We find that the dissociation route for Y_2_@C_79_N is CN-elimination (Supplementary Fig. 16), in contrast to C_2_-loss with retention of N for the Y_3_N@C_80_ endohedral nanocluster. These results are consistent with empty cage N-containing heterofullerenes^44^. Thus, the present gas-phase dissociation studies are shown to be a structural diagnostic for identification of clusterfullerenes and endohedral heterofullerenes.

The mechanistic uniqueness of Sc_3_N@*I*_h_-C_80_ is clearly established in this work by its lack of growth into larger species, whereas Y_3_N@*I*_h_-C_80_ clearly diverges from that trend and can grow into cages that exceed 100 carbon atoms in size. These results also support the assignment of a high-symmetry cage, such as *I*_h_-C_80_, as a major contributing isomer for the magic numbered Sc_3_N@C_80_ nanocluster formed from bulk starting materials by laser synthesis, although other isomers should be produced to a lesser extent. Pyramidalization of carbon atoms takes place when a metal is coordinated to the [5,6] carbon atoms for Sc_3_N@C_80_ compared to the center of a hexagon for Y_3_N@C_80_^45^. Consequently, the pyrene-type carbon atoms in Y_3_N@C_80_ are more strained and may be more reactive to carbon insertion reactions. It is noteworthy that the M_3_N-encapsulated product cages shown in Fig. 4 must be lower symmetry than the *I*_h_-C_80_ precursor. Interestingly, the small non-IPR Sc_3_N@*D*_3_-C_68_ compound that contains three sets of fused pentagons appears to be somewhat less reactive in the bottom-up growth scheme than Y_3_N@*I*_h_-C_80_. That result distinguishes how the transfer of six electrons and an optimally sized M_3_N cluster significantly renders non-IPR cages less reactive through bottom-up mechanisms. Those observations further account for the different formation trends for Sc_3_N@C_2n_ and Y_3_N@C_2n_ from the original doped graphite.

## Discussion

We propose that high-symmetry clusterfullerenes primarily self-assemble through a bottom-up mechanism by simple vaporization of graphite. Online chemical sampling of laser vaporized doped graphite reveals an initial nucleation step, whereby M_3_N nucleates formation of the smallest possible cage(s). For example, Sc_3_N bypasses cages smaller than C_62_, whereas the larger Y_3_N cluster must initially nucleate cages of C_74_ or larger under our conditions. Those results clarify that formation initiates through bottom-up processes that exhibit a cluster size effect on cage selection. Avoidance of *I*_h_-C_60_ in the bottom-up mechanism and thus the many other small “bottleneck” cages, C_28_, C_36_, C_44_, C_50_, in reaction paths that severely limit growth to medium-sized conventional metallofullerenes, M@C_2n_^23, 46^, enable clusterfullerene cages in the solvent-extractable ~C_70_–C_100_ region to form in inherently higher abundance. Further, that mechanistic property allows breakthrough access to the next possible icosahedral cage, *I*_h_-C_80_, which may then act as a mechanistic bottleneck in formation for group 3 NCFs, and, in particular, should permit Sc_3_N@*I*_h_-C_80_ to accumulate in bottom-up reaction paths and thus explains its high-yield formation.

Our bottom-up model for NCF formation is further experimentally tested by extensive investigations on specified isomers of the group 3-based clusterfullerenes, Sc_3_N@*D*_3_-C_68_, Sc_3_N@*I*_h_-C_80_, Sc_3_N@*D*_5h_-C_80_, and Y_3_N@*I*_h_-C_80_ by exposure to graphite vapor under characteristic physicochemical synthetic conditions. Fused pentagon-containing Sc_3_N@C_68_ unambiguously grows into Sc_3_N@C_80_, which exhibits an enhanced abundance in agreement with the proposal that medium-sized Sc_3_N@C_2n_ from bulk doped graphite primarily form through a bottom-up mechanism. The archetypal clusterfullerene, Sc_3_N@*I*_h_-C_80_, is observed to be rather inert to further bottom-up growth, which is consistent with the assignment of *I*_h_-C_80_ for Sc_3_N@C_80_ from doped graphite and from the explicit growth of Sc_3_N@*D*_3_-C_68_ in this work. Theoretical investigations show that cage transformations from *D*_3_-C_68_ cage into *I*_h_-C_80_ can occur by a total of six C_2_ insertions and only two to three SW rearrangements. Heptagon-containing and classical cages are predicted to be involved in these transformations. The *D*_5h_-C_80_ isomer can self-assemble without any SW rearrangements through six direct C_2_ insertion events.

Substitution of Y_3_N in the *I*_h_-C_80_ cage dramatically alters the molecular behavior of *I*_h_-C_80_ in graphite vapor and Y_3_N@C_80_ readily grows into clusterfullerenes that exhibit 100 carbon atom cages. That cluster-cage size effect further explains the shift to larger cages in formation distributions for Sc_3_N@C_2n_ to Y_3_N@C_2n_ from doped graphite. Unexpectedly, we find that NCFs can transform into five-atom cluster entrapped cages that presumably result from intramolecular cage reactions that take place during bottom-up growth of Sc_3_N@C_68_. For example, endohedral species with a chemical composition of Sc_3_NC_81_ are formed that may be the origin of five-atom cluster encapsulated, Sc_3_NC@C_80_, and thus offers another mechanistic route to clusterfullerene formation through bottom-up growth paths. We do not observe formation of M_3_NC_81_ by growth of the larger Sc_3_N@*I*_h_-C_80_, Sc_3_N@*D*_5h_-C_80_, or Y_3_N@*I*_h_-C_80_ clusterfullerenes NCFs under the present conditions, suggesting that cluster interaction with the cage during bottom-up growth could be crucial to its formation.

In conclusion, through in situ investigations of clusterfullerenes synthesized by laser vaporization of doped graphite and study of discrete NCF compounds, combined with extensive theoretical investigations, we disclose that bottom-up self-assembly reactions are responsible for synthesis of high-symmetry carbon cages that encapsulate metallic nitride clusters. We propose that these intrinsic chemical processes are a fundamental property of carbon under harsh conditions typical of synthesis and will help tackle the challenge of carbon nanostructure formation for other hybrid carbon allotropes and should be useful for synthesis of new carbon-based cluster compounds. We note that it is possible that a “local” C_2_-loss event may occur in a “global” bottom-up path or in the solid state after formation, and therefore should also be considered to comprehensively describe fullerene formation. Future in situ studies of doped graphite, combined with the analysis of distinct clusterfullerenes, are now possible for compounds that encapsulate other endohedral clusters (e.g., metal carbides, sulfides, oxides, etc.)^47–53^ and possess various structural motifs, which should facilitate the exploration of new forms of encapsulated nanocarbon materials and their fundamental self-assembly processes.

## Methods

### Clusterfullerenes from doped graphite

Doped graphite starting materials are produced by physical mixing of graphite (99.9995%, 2−15 μm), scandium oxide or yttrium oxide (99.9%), and melamine (99%)^23^. The doped graphite material is then molded into a 12.7-mm rod by compression of the mixture. Doped graphite rods are comprised of 10 atom % metal, with a 1:2 ratio for metal:C_3_H_6_N_6_.

### Reactivity of cluster-encapsulated cages in graphite vapor

Macroscopic samples of metallic NCFs are synthesized by arc discharge. Isomerically pure samples of Sc_3_N@*I*_h_-C_80_ and Sc_3_N@*D*_3_-C_68_ were purified by use of non-chromatographic methods^54^. Y_3_N@*I*_h_-C_80_ was purified by multi-stage HPLC. Isomerically pure NCF was then uniformly applied to the surface of a pristine 12.7-mm graphite rod (99.9995%, 2−15 μm) for nanocarbon reaction studies by use of a Nd:YAG (532 nm, 10 mJ) pulsed laser cluster source^23, 25^. NCFs were individually applied to a quartz rod for high energy, direct laser ablation without exposure to carbon plasma.

### Cluster source and 9.4 T FT-ICR mass spectrometry

Self-assembly reaction experiments are analyzed with a custom-built FT-ICR mass spectrometer based on a 9.4 T superconducting magnet and performed with positive ions produced by a pulsed laser cluster source^23, 24^. Evaporation of a doped graphite stationary rod (12.7-mm diameter) is achieved by a single laser shot fired from a Nd:YAG (532 nm, 3–5 ns pulse width, 1.5-mm beam diameter) in conjunction with the opening of a pulsed valve (800 ms duration) to admit He flow over the sample. Carbon vapor produced then enters a channel 4 mm in diameter and ~8.5 mm in length. The laser is fired ~2 ms after opening of the pulsed valve for evaporation of doped graphite samples and ~4 ms for Sc_3_N@C_68_, Sc_3_N@*I*_h_-C_80_, Sc_3_N@*D*_5h_-C_80_, and Y_3_N@*I*_h_-C_80_-coated graphite samples. Ions accumulated by ten individual laser and helium pulse events are transported to an open cylindrical ion trap (70-mm diameter, 212-mm long, aspect ratio ~2). The ions are accelerated to a detectable cyclotron radius by a broadband frequency sweep excitation (260 Vp-p, 150 Hz µs^−1^, 3.6 down to 0.071 MHz) and subsequently detected as the differential current induced between two opposed electrodes of the ICR cell. Each of the acquisitions is Hanning-apodized and zero-filled once before fast Fourier transform and magnitude calculation^23^. Up to ten time-domain acquisitions are averaged. The experimental event sequence is controlled by a modular ICR data acquisition system. Ions are further probed by CID.

### Computational details

Amsterdam Density Functional code (ADF2012) was used for the electronic structure calculations^55^. The electronic density was provided by the local density approximation by use of Becke’s gradient corrected exchange functional, and Vosko, Wilk, Nusair parametrization for correlation, corrected with Perdew’s functional (BP86). Electrons for all the atoms were described with Slater-type basis functions of triple-ζ + polarization quality. We have included scalar relativistic corrections by means of the zeroth-order regular approximation formalism. All Sc_3_N@C_2n_ calculations have also been performed including dispersion corrections. We used the CaGe program to generate fullerenes and Schlegel diagrams.

### Data availability

Data that support the findings of this study are available within the paper and its supplementary information files, and available from the corresponding author upon request. A data set collection of computational results is available from the online ioChem-BD repository and can be accessed via https://doi.org/10.19061/iochem-bd-2–16, where all intermediates described in Fig. 3 and Supplementary Fig. 10 can be found.

## Electronic supplementary material

Supplementary Information

Peer Review File
