# Supplementary material for: Transformation of doped graphite into cluster-encapsulated fullerene cages
Source: Nat Commun. 2017 Oct 31;8:1222. doi: 10.1038/s41467-017-01295-9 (PMC5663703; doi:10.1038/s41467-017-01295-9)
Supplement: Supplementary file 1 — Supplementary Information [file 41467_2017_1295_MOESM1_ESM.pdf]

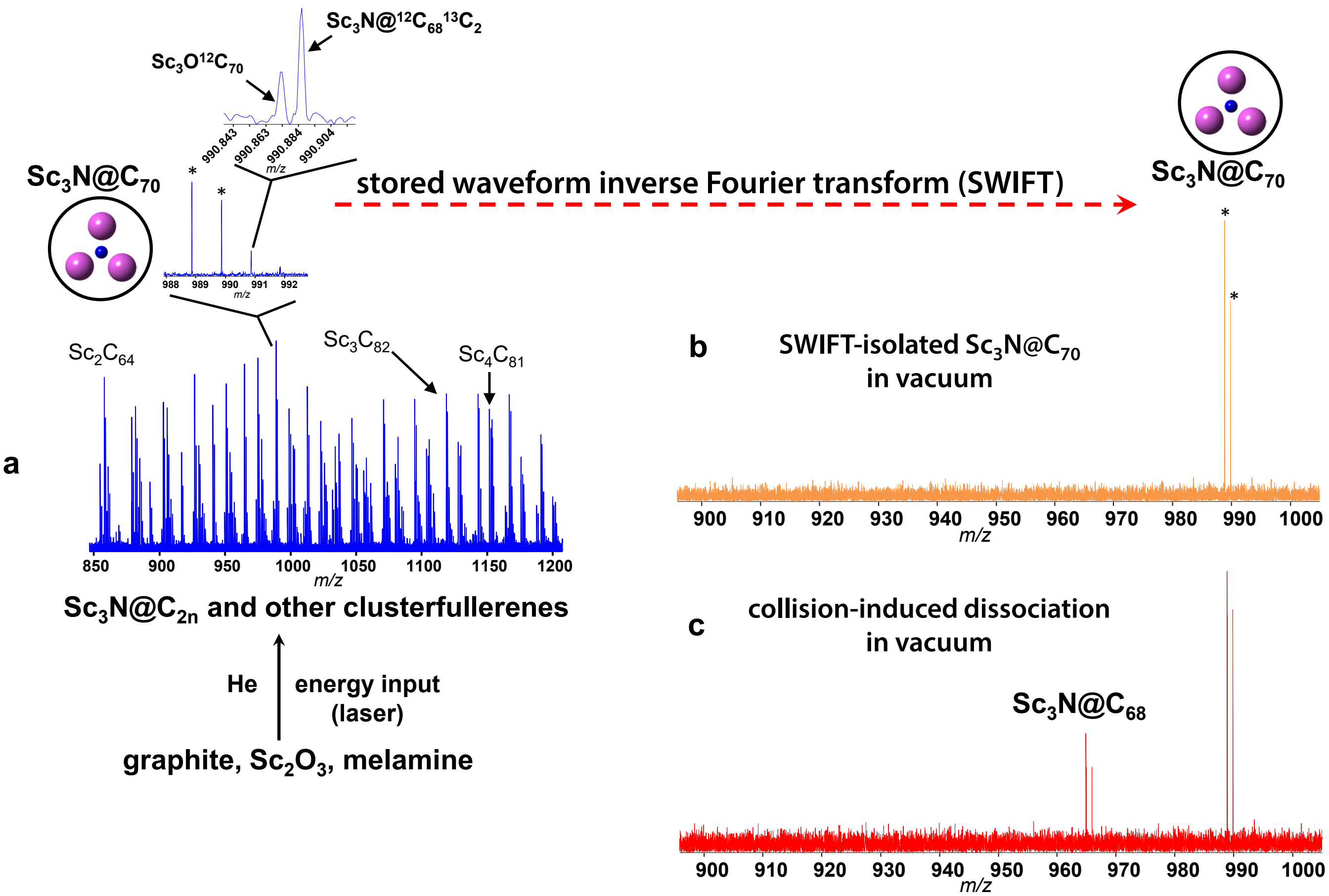

Supplementary Figure 1. (**a**) Clusterfullerenes (positive ions) generated by laser vaporization of graphite, Sc_2O_3, and melamine. (**b**) SWIFT-isolated $Sc_3N@C_{70}$ formed from those bulk starting materials. (**c**) Sustained off-resonance irradiation collision-induced dissociation (SORI-CID) for $Sc_3N@C_{70}$. Analysis by the present 9.4 T FT-ICR mass spectrometer provides ultrahigh resolution and high mass accuracy, permitting unambiguous detection of all molecular ions. $Sc_3N@C_{70}$ is confirmed to exhibit a nitride clusterfullerene (endohedral metallofullerene) structure because C_2-elimination with retention of the internally bound cluster, Sc_3N, is the only dissociation process observed when highly thermally excited in the gas phase in vacuo. Two isotopologues* of $Sc_3N@C_{70}$, namely, $Sc_3N@{}^{12}C_{70}$ and $Sc_3N@{}^{12}C_{69}{}^{13}C$, are isolated in this experiment to exclude the molecular ion, $Sc_3O^{12}C_{70}$, which has the same nominal mass as $Sc_3N@{}^{12}C_{68}{}^{13}C_2$. Note: all mass spectra in this paper correspond to positive ions.

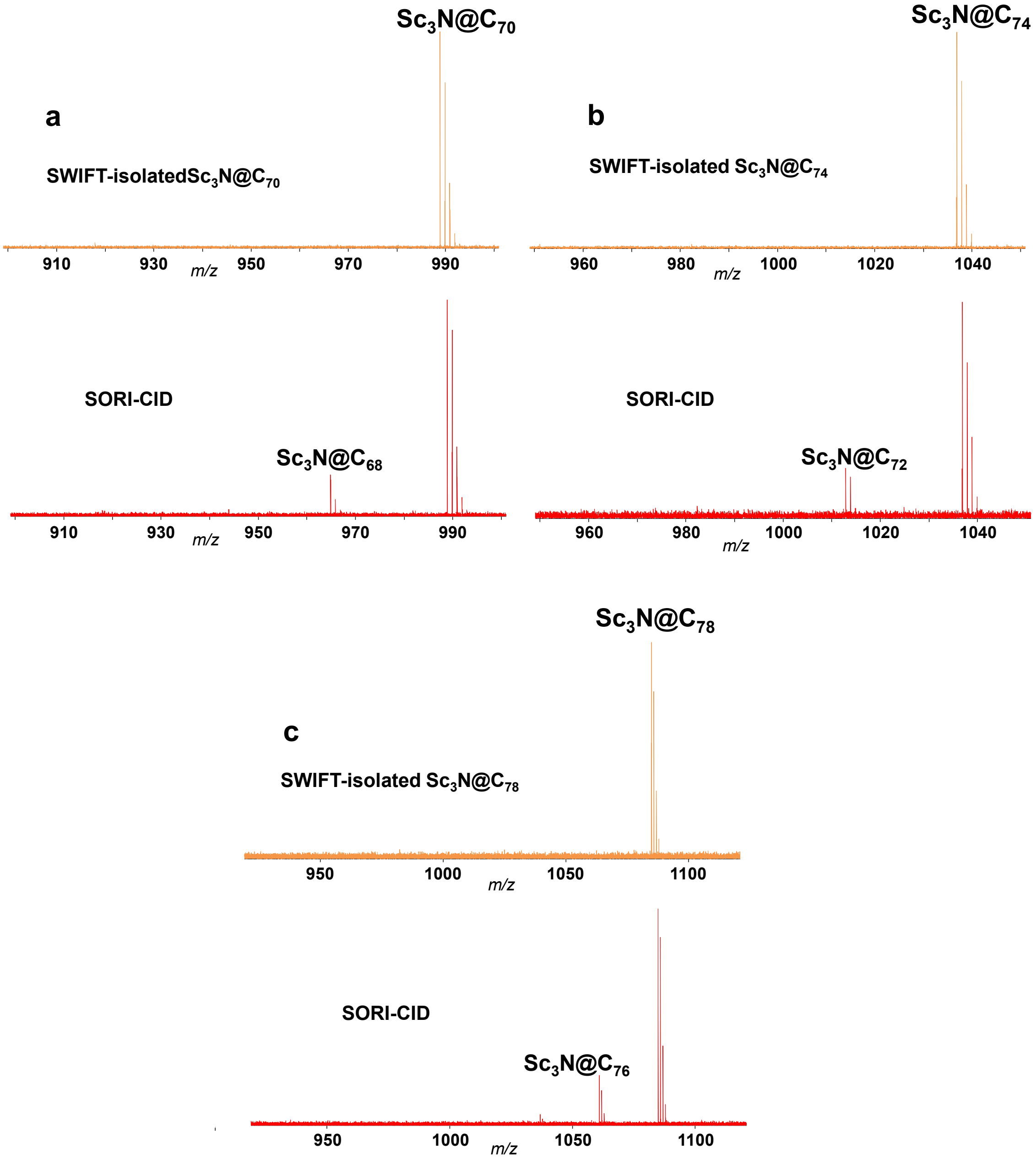

Supplementary Figure 2. SWIFT-isolation and SORI-CID product ion mass spectra for $Sc_3N@C_{70}$ (**a**), $Sc_3N@C_{74}$ (**b**), and $Sc_3N@C_{78}$ (**c**), which are all bottom-up self-assembly products formed by exposure of $Sc_3N@D_3\text{-}C_{68}$ to carbon vapor in He. C_2-elimination with retention of Sc_3N is the only process observed, thus confirming these growth products are metallic nitride clusterfullerenes.

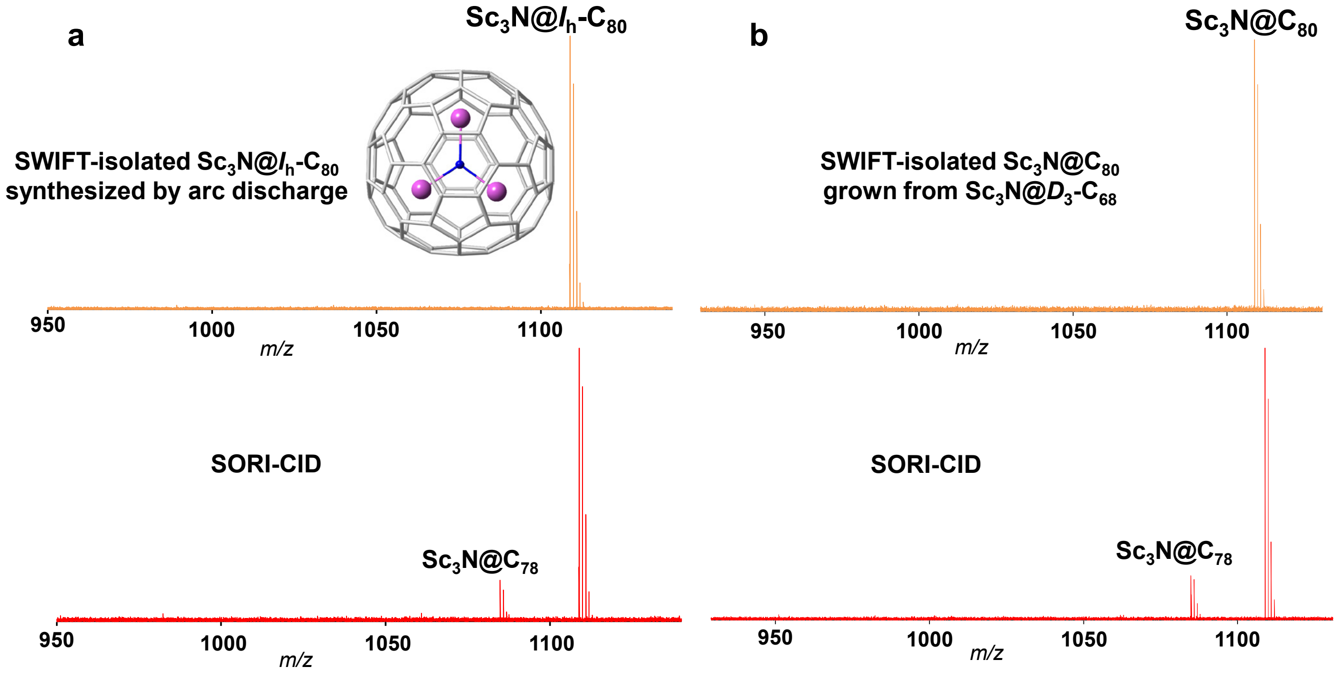

Supplementary Figure 3. SWIFT and SORI-CID product ion spectra for (**a**) pre-existing, $Sc_3N@I_h$-C_{80} and (**b**) $Sc_3N@C_{80}$ formed by the growth of $Sc_3N@D_3$-C_{68} in carbon plasma**.** Both experiments are performed under identical collision-induced dissociation conditions in the gas phase. We emphasize that two sources of $Sc_3N@C_{80}$ are used for dissociation analysis in this figure, (i) isomerically pure, ***pre-existing*** $Sc_3N@I_h$-C_{80} material produced by the arc plasma discharge method, shown in (**a**)**,** and (ii) $Sc_3N@C_{80}$ ***grown*** *in situ* from $Sc_3N@D_3$-C_{68} in carbon plasma, shown in (**b**).

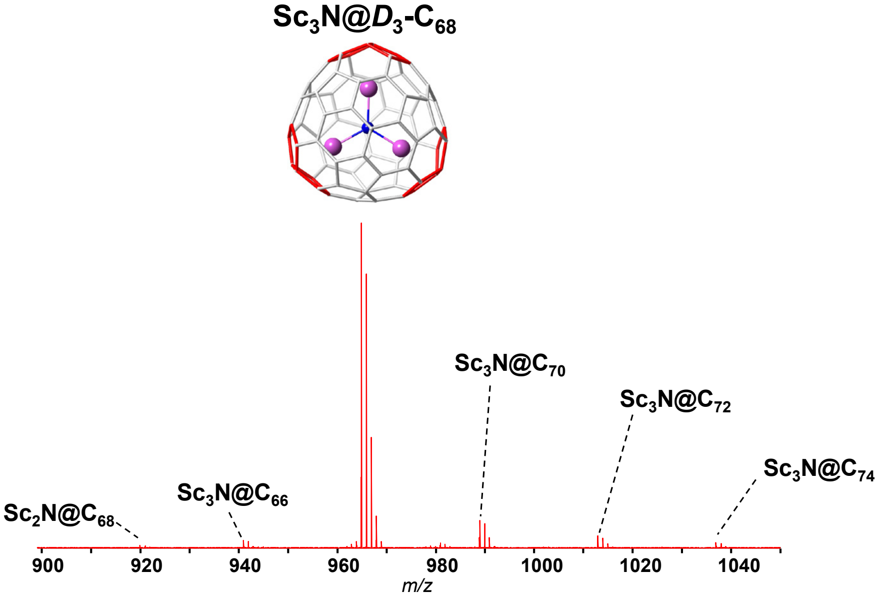

Supplementary Figure 4. Laser vaporized $Sc_3N@\textit{D}_3\text{-}C_{68}$ at high energy, without the presence of carbon vapor. The laser fluence (10 mJ per pulse) is identical to that for all carbon vapor exposure studies for $M_3N@C_{2n}$ and vaporization of bulk starting materials (i.e., graphite, metal oxide, and melamine) to facilitate comparisons. Carbon insertion reactions are still the preferred process at low carbon densities, evidenced by the formation of $Sc_3N@C_{70}$ and even larger $Sc_3N@C_{2n}$, whereas C_2-elimination is scarcely detectable under the present conditions. The Sc-loss product, $Sc_2N@C_{68}$, is also observed at very low relative abundance.

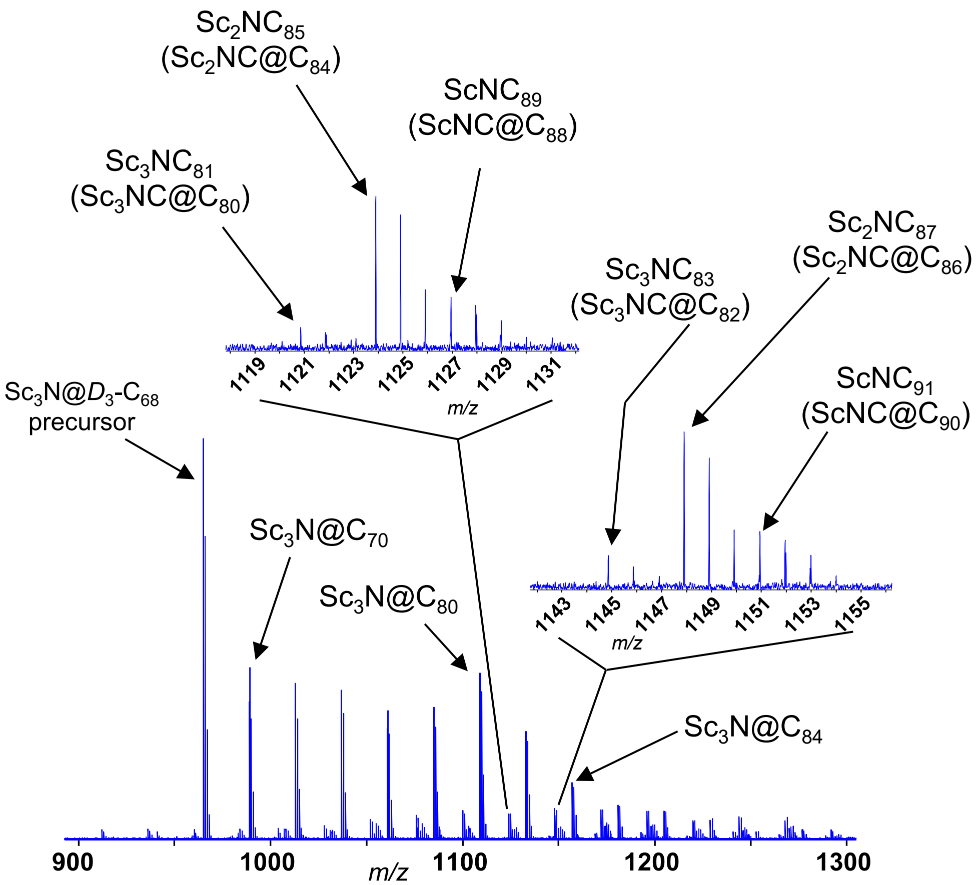

Supplementary Figure 5. Mass scale expansions of other families of clusterfullerene molecular ions generated by exposure of $Sc_3N@D_3$-C_{68} to carbon vapor from graphite. The smallest species observed for the Sc_3NC_n family (C_n = odd number of carbon atoms) is Sc_3NC_{81}. Possible endohedral structures are in parenthesis.

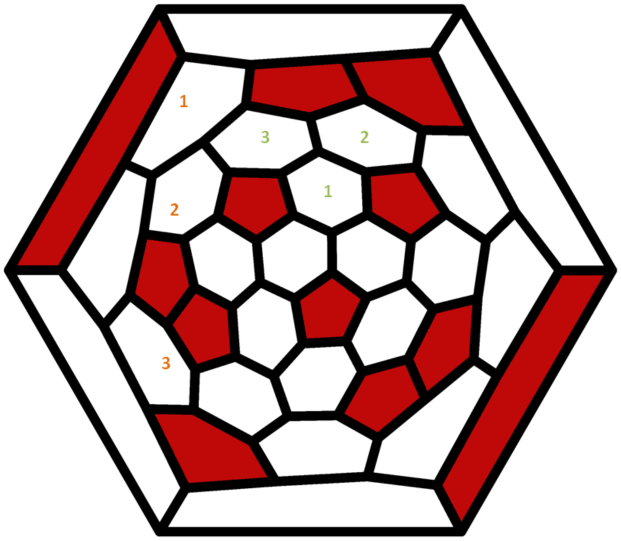

D_3-C_{68}(6140)

Isomer	C_{70}^{6-}
C_{2v} (7854)	0
C_2 (7892)	16.1
Hept formation	
ins1	57.9
ins2	25.5
ins3	47.5
CF formation	
ins1	36.1
ins2	36.4
ins3	13.8

Supplementary Figure 6. Schlegel diagram of D_3-C_{68}(6140) with C_2 insertion sites identified that can form the heptagon (hept) or the classical fullerene (CF) C_{70} structures described in this work. Red numbers indicate the formation of the heptagon structure and green the classical fullerene. The energies of the structures formed are shown in kcal·mol^{-1}. Note that 'Ins' is the abbreviation for the inserted position according to the Schlegel diagram.

a

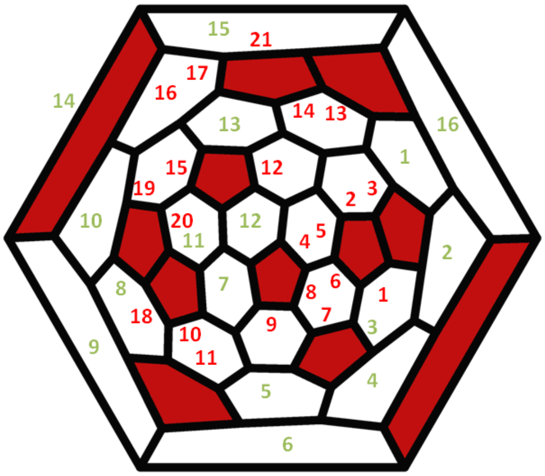

C_{70}(7886)

Isomer	C_{72}^{6-}
C_s(10528)	0
Hept formation	
ins1	65.7
ins2	24.2
ins3	65.4
ins4	68.4
ins5	41.1
ins6	61.9
ins7	98.0
ins8	73.9
ins9	72.4
ins10	50.6
ins11	76.3
ins12	40.4
ins13	63.5
ins14	40.7
ins15	83.8
ins16	87.6
ins17	47.7
ins18	65.8
ins19	52.9
ins20	69.0
ins21	71.5

Isomer	C_{72}^{6-}
CF formation	
ins1	16.1
ins2	49.0
ins3	52.6
ins4	59.8
ins5	45.6
ins6	58.7
ins7	24.6
ins8	60.4
ins9	57.2
ins10	30.3
ins11	66.0
ins12	44.9
ins13	24.3
ins14	60.8
ins15	57.9
ins16	48.7

b

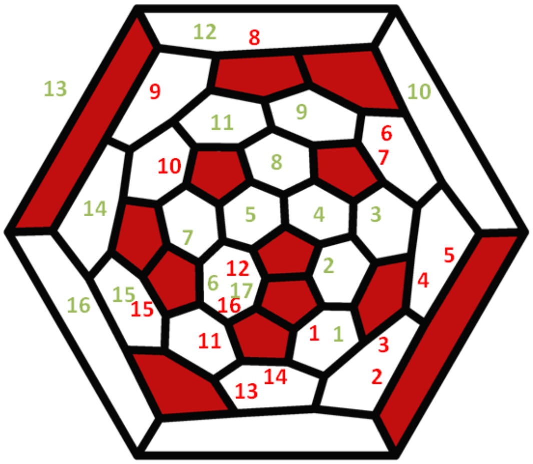

C_{70}(hept)

Isomer	C_{72}^{6-}
C_s(10528)	0
Hept formation	
ins1	75.1
ins2	64.4
ins3	81.8
ins4	74.2
ins5	87.5
ins6	61.3
ins7	88.3
ins8	80.5
ins9	90.1
ins10	92.5
ins11	55.0
ins12	59.3
ins13	65.6
ins14	77.4
ins15	101.0
ins16	53.5

Isomer	C_{72}^{6-}
CF formation	
ins1	63.4
ins2	96.3
ins3	67.1
ins4	66.3
ins5	41.6
ins6	16.3
ins7	61.0
ins8	74.2
ins9	65.7
ins10	47.6
ins11	53.4
ins12	66.5
ins13	77.3
ins14	50.8
ins15	69.2
ins16	61.0
ins17	0.0

Supplementary Figure 7. Schlegel diagram of (**a**) C_{70}(7886) and (**b**) C_{70}(hept) with C_2 insertion sites identified that can form the heptagon (hept) or the classical fullerene (CF) C_{72} structures described in this work. Red numbers indicate the formation of the heptagon structure and green the classical fullerene. The energies of the structures formed are shown in kcal·mol^{-1}. Note that 'Ins' is the abbreviation for the inserted position according to the Schlegel diagram.

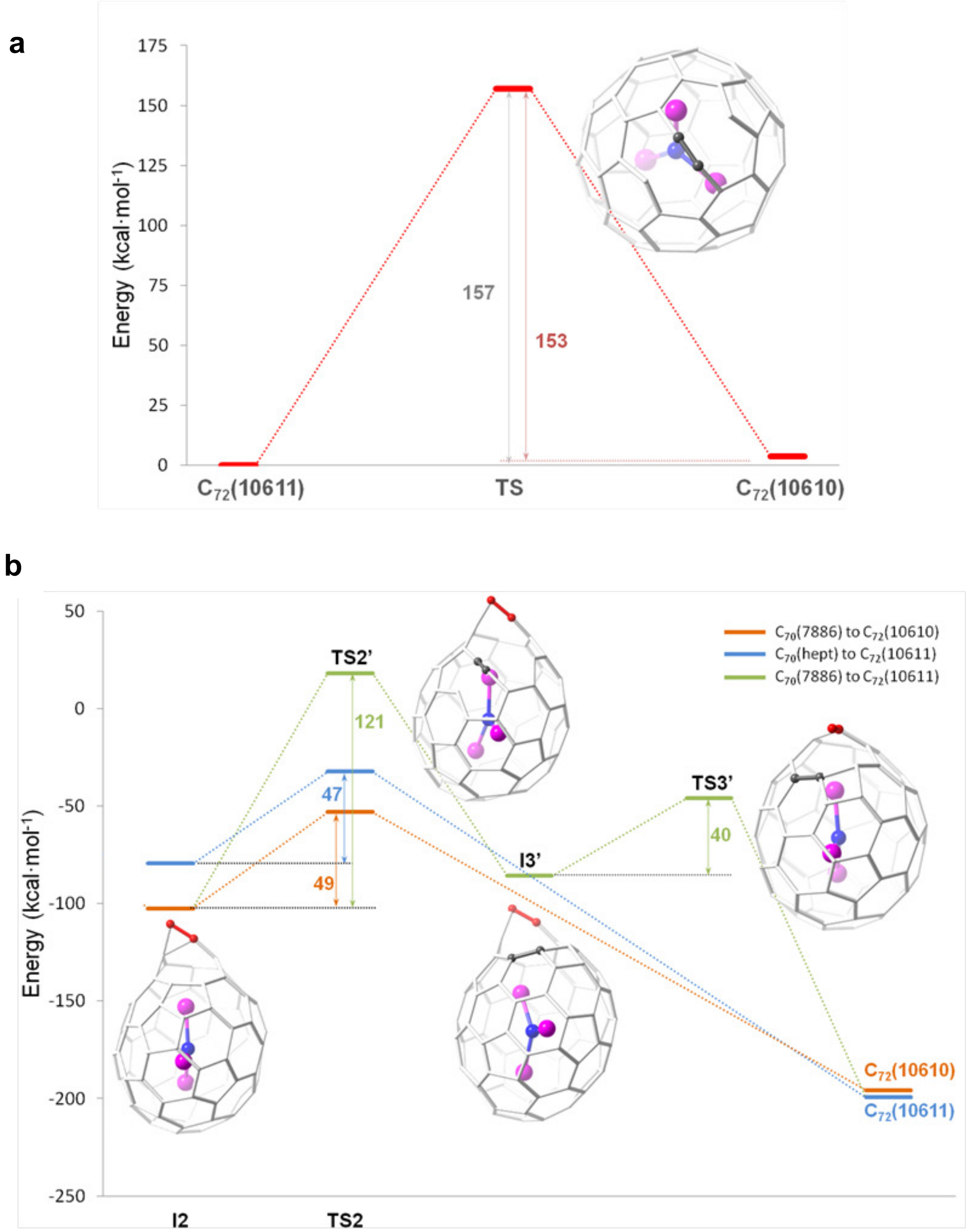

Supplementary Figure 8. C_2 rearrangements for $Sc_3N@C_{72}$ formed from $Sc_3N@C_{70}$ during carbon insertion with conventional SW in closed fullerenes (**a**) and 'open structures' (**b**). The energy barrier of the simple SW transformation is around 153-157 kcal·mol^{-1}. We have also studied the SW rearrangement mechanism that relates **I2** of $Sc_3N@C_{70}$(7886) + C_2 to $Sc_3N@C_{72}$(10611). It is not a concerted mechanism because two transition states are found, **TS2'** and **TS3'**. The energy barrier that connects **I2** and **I3'**, i.e. when the rearrangement takes place in the open structure **I2**, is 120.7 kcal·mol^{-1}, and is lower than the direct SW found previously (~150-160 kcal·mol^{-1}). The last step is the closure of the cage; **I3'** is related to $Sc_3N@C_{72}$(10611) by a transition state **TS3'** that has a lower energy barrier (39.8 kcal·mol^{-1}) than **TS2** (the transition state from direct C_2 insertion to C_{70}(hept) without a SW transformation). Thus, the isomerization process in **I2** structures could be energetically more feasible than for closed cages.

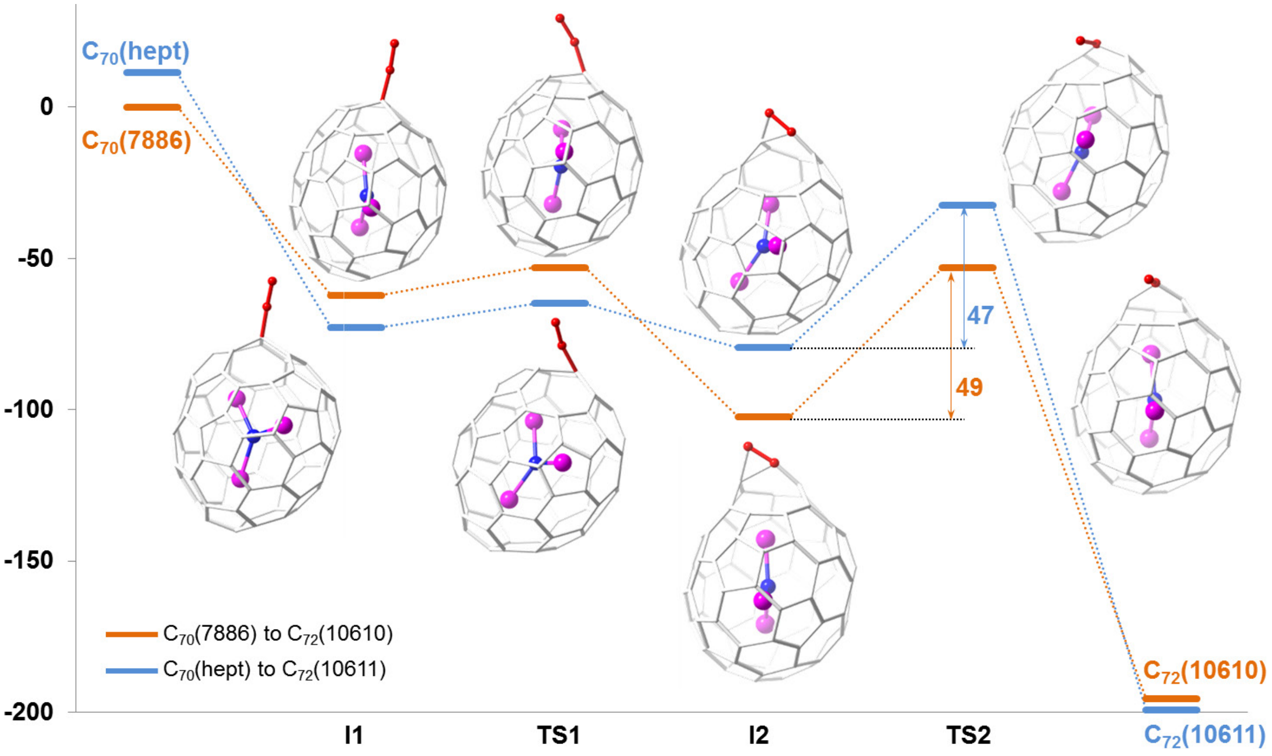

Supplementary Figure 9. Our computations suggest that the C_2 unit reacts with a [5,6,6] atom of a hexagon in C_{70}, forming intermediate, **I1**, in which a single atom of the C_2 molecule is attached to the cage. The second intermediate, **I2**, displays stability and contains a heptagonal ring or an eight-membered ring from C_{70}(hept). The last step involves complete C_2 integration or cage closure, whereby two pentagons are formed as a result of growth from C_{70}(7886) or a pentagon and hexagon pair from C_{70}(hept). The energetic barrier to overcome this last step is 50 kcal·mol^{-1}, which is achievable at the typical temperature range of synthesis (>1000 K). The associated Gibbs free energy is computed to be approximately -110 kcal·mol^{-1}. Therefore, C_2 insertion is a favorable exothermic process. These results are consistent with C_2 insertion events recently proposed for small titanium-based mono-metallofullerenes.

a

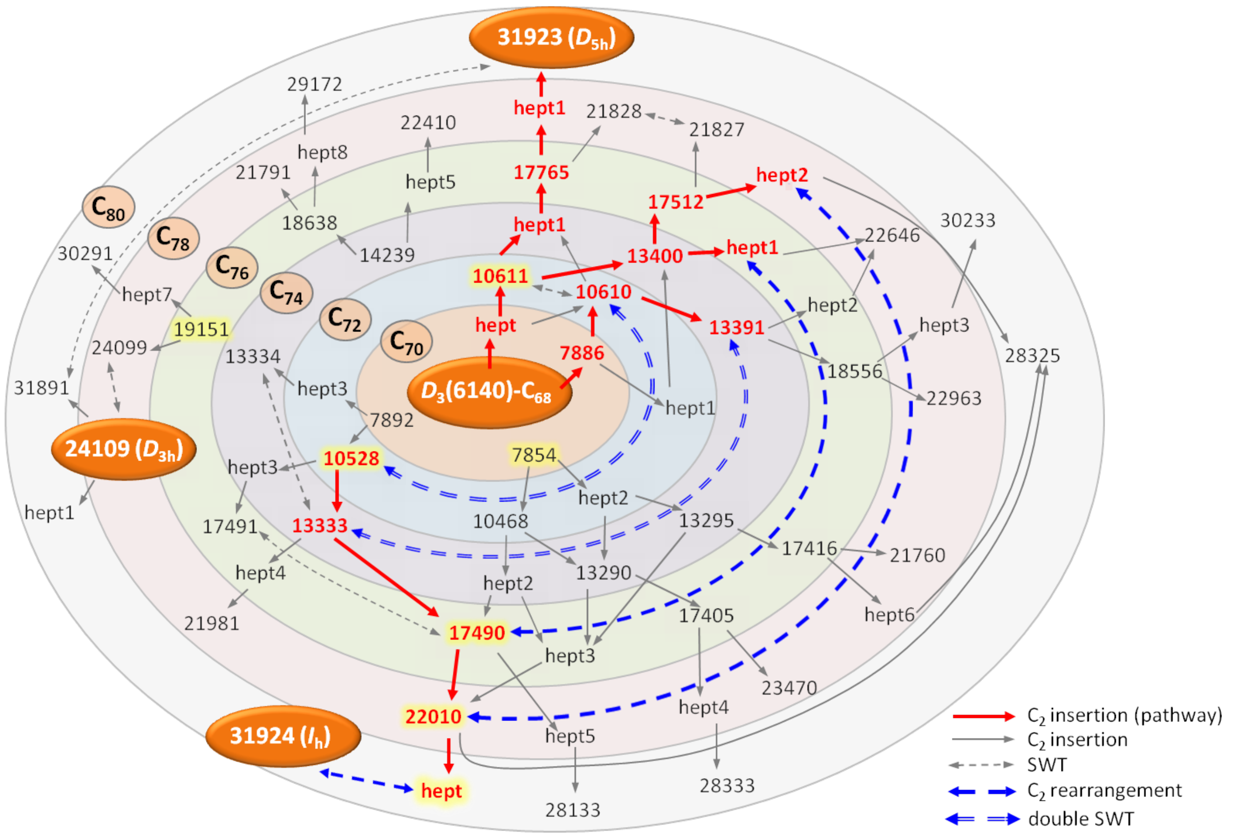

b

Pathway i: D_3-C_{68}(6140) → C_{70}(hept) → C_{72}(10611) → C_{74}(13400) → C_{76}(17512) → C_{78}(hept2) ⇔ C_{78}(22010) → C_{80}(hept) ⇔ I_h-C_{80}(31924)

Pathway ii: D_3-C_{68}(6140) → C_{70}(hept) → C_{72}(10611) → C_{74}(13400) → C_{76}(hept1) ⇔ C_{76}(17490) → C_{78}(22010) → C_{80}(hept) ⇔ I_h-C_{80}(31924)

Pathway iii: D_3-C_{68}(6140) → C_{70}(7886) → C_{72}(10610) → C_{74}(13391) ⇔⇔ C_{74}(13333) → C_{76}(17490) → C_{78}(22010) → C_{80}(hept) ⇔ I_h-C_{80}(31924)

Pathway iv: D_3-C_{68}(6140) → C_{70}(7886) → C_{72}(10610) ⇔⇔ C_{72}(10528) → C_{74}(13333) → C_{76}(17490) → C_{78}(22010) → C_{80}(hept) ⇔ I_h-C_{80}(31924)

Pathway v: D_3-C_{68}(6140) → C_{70}(hept) → C_{72}(10611) → C_{74}(hept1) → C_{76}(17765) → C_{78}(hept1) → D_{5h}-C_{80}(31923)

Supplementary Figure 10. (**a**) Schematic diagram of routes for the transformation of D_3-C_{68} into C_{80} cages in a bottom-up growth mechanism. Bold arrows represent pathways that yield I_h-C_{80} from D_3-C_{68} by simple C_2 insertions (red) and C_2 structural rearrangements (blue). Known endohedral cage isomers that have been structurally characterized are highlighted in yellow. (**b**) Selected pathways that link D_3-C_{68}(6140) to I_h-C_{80}(31924) and D_{5h}-C_{80}(31923) by carbon insertions via low energy isomers with a minimal number of C_2 structural rearrangements. The symbol → denotes a C_2 insertion and ⇔ identifies a C_2 rearrangement.

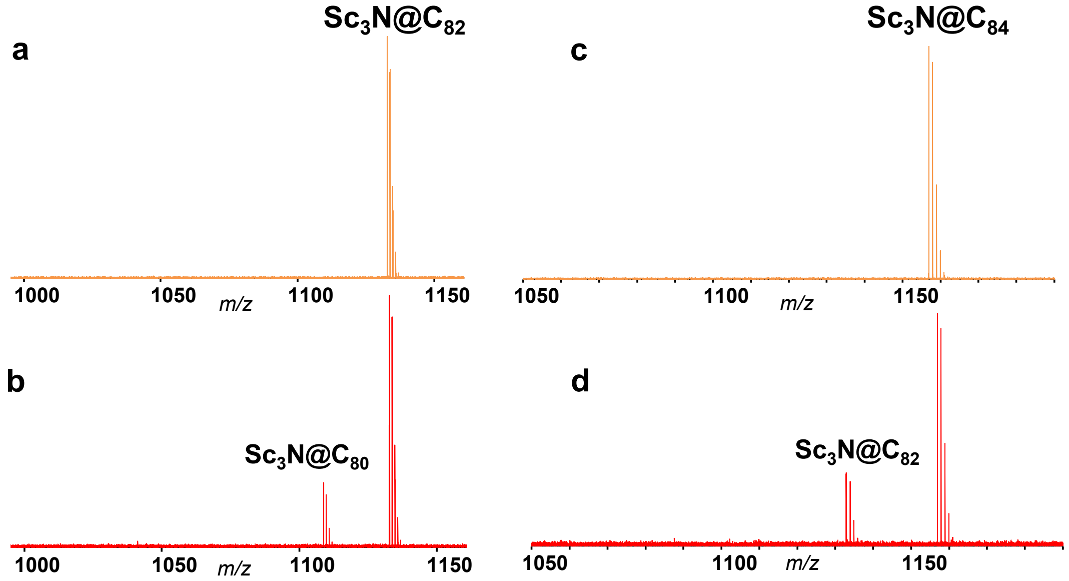

Supplementary Figure 11. (**a**) SWIFT-isolated $Sc_3N@C_{82}$ formed by C_2 insertion into the $Sc_3N@I_h$-C_{80} precursor compound. (**b**) Sustained off-resonance irradiation collision induced dissociation (SORI-CID) product ion mass spectrum of isolated $Sc_3N@C_{82}$ in an ultrahigh vacuum. (**c**) SWIFT-isolated $Sc_3N@C_{84}$ formed by laser vaporization of a $Sc_3N@I_h$-C_{80} coated graphite rod and (**d**) SORI-CID. These growth product species are unambiguously demonstrated to exhibit endohedral metallic nitride structures. Internally bound elements, Sc_3N, remain trapped inside the cage when high thermally excited, whereas exohedral elements attached to the cages would readily dissociate.

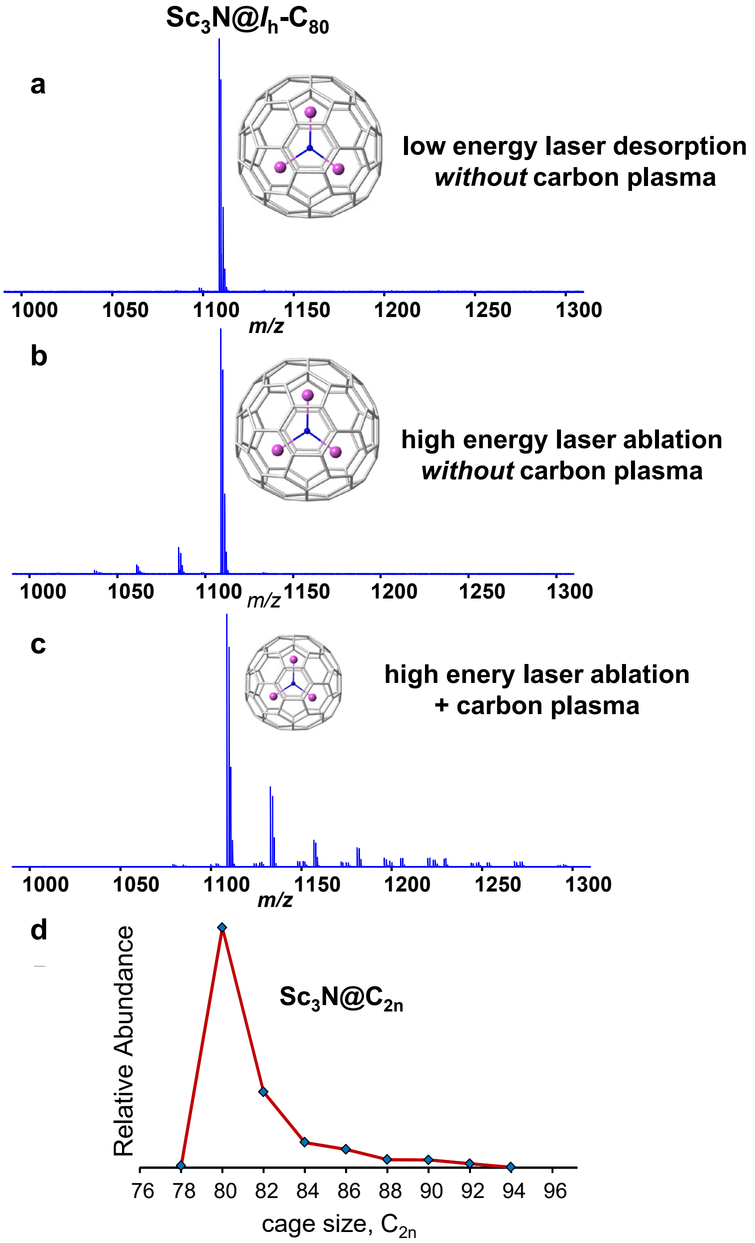

Supplementary Figure 12. (**a**) Laser irradiation of $Sc_3N@I_h$-C_{80} coated onto a quartz rod at low laser fluence (1-2 mJ/pulse). (**b**) Laser irradiation of $Sc_3N@I_h$-C_{80} on a quartz rod at high laser fluence (10 mJ/pulse), without exposure to carbon vapor. (**c**) Larger clusterfullerenes formed after exposure of $Sc_3N@I_h$-C_{80} to carbon plasma by laser ablation (10 mJ/pulse) of a $Sc_3N@I_h$-C_{80} coated graphite rod. (**d**) Growth trend of $Sc_3N@C_{2n}$ formed from the $Sc_3N@C_{80}$ precursor after exposure to carbon vapor. Little fragmentation (C_2-loss) for $Sc_3N@I_h$-C_{80} is observed after laser ablation at high energy without carbon vapor (i.e., low carbon density), whereas high energy laser ablation of $Sc_3N@I_h$-C_{80} in the presence of graphite vapor results in bottom-up formation of $Sc_3N@C_{82}$ in abundance, with $Sc_3N@_{84}$ to $Sc_3N@_{94}$ observed at lower abundance.

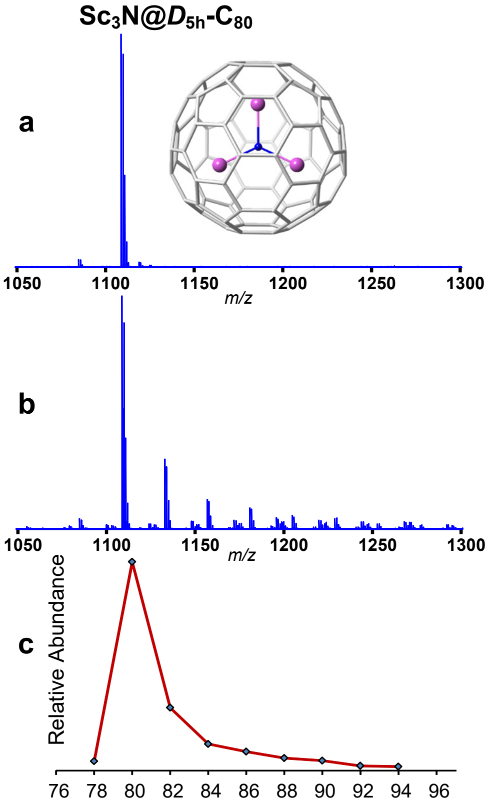

Supplementary Figure 13. (**a**) Laser irradiation of $Sc_3N@D_{5h}$-C_{80} coated onto a quartz rod at low laser fluence (1-2 mJ/pulse). (**b**) Larger clusterfullerenes formed after exposure of $Sc_3N@D_{5h}$-C_{80} to carbon plasma by laser ablation (10 mJ/pulse) of a $Sc_3N@D_{5h}$-C_{80} coated graphite rod. (**c**) Growth trend of $Sc_3N@C_{2n}$ formed from the $Sc_3N@C_{80}$ precursor after exposure to carbon vapor. Little fragmentation (C_2-loss) for $Sc_3N@D_{5h}$-C_{80} is observed after laser ablation at high energy without carbon vapor (i.e., low carbon density), whereas high energy laser ablation of $Sc_3N@D_{5h}$-C_{80} in the presence of graphite vapor results in bottom-up formation of $Sc_3N@C_{82}$ in abundance, with $Sc_3N@_{84}$ to $Sc_3N@_{94}$ observed at lower abundance.

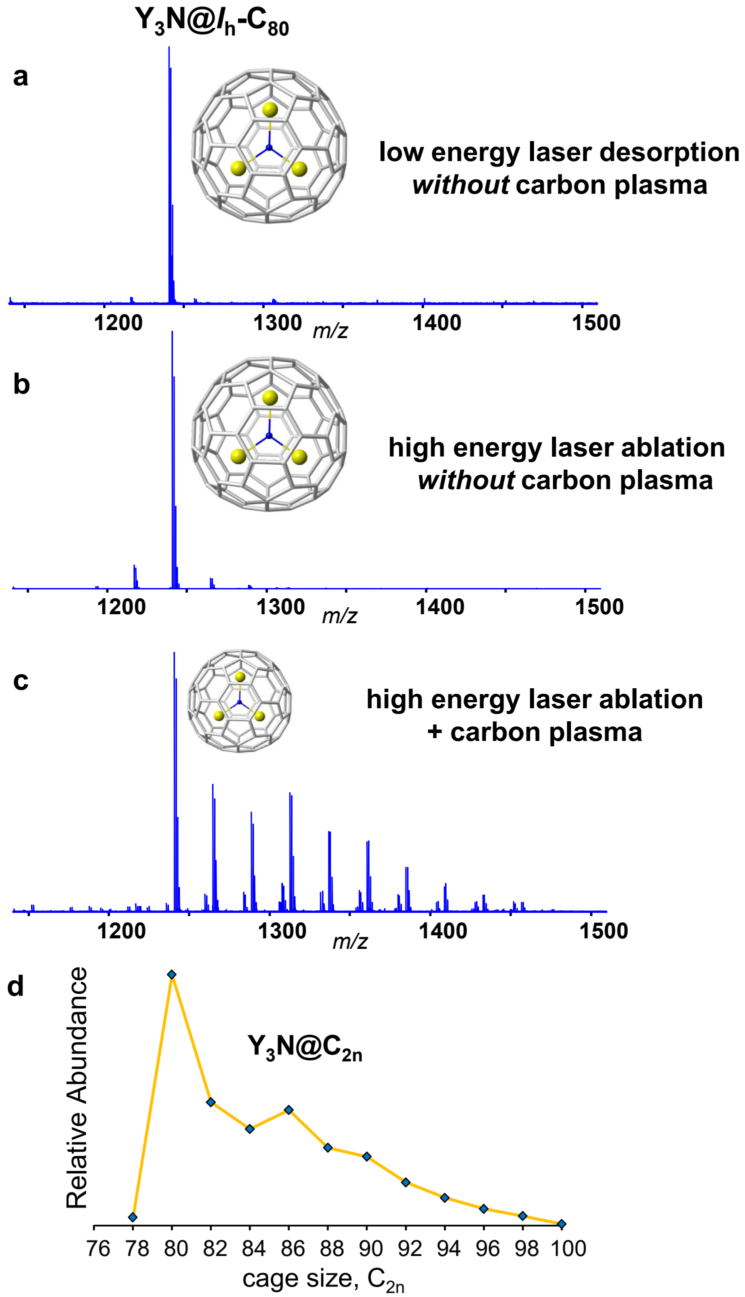

Supplementary Figure 14. (**a**) Laser irradiation of isomerically pure $Y_3N@I_h\text{-}C_{80}$ coated onto a quartz rod at low laser fluence (1-2 mJ/pulse). (**b**) Laser irradiation of $Y_3N@I_h\text{-}C_{80}$ at high laser fluence (10 mJ/pulse), without exposure to carbon vapor. (**c**) Larger clusterfullerenes formed after exposure of $Y_3N@I_h\text{-}C_{80}$ to carbon plasma by laser ablation (10 mJ/pulse) of a $Y_3N@I_h\text{-}C_{80}$ coated graphite rod. (**d**) Growth trend for $Y_3N@C_{2n}$ formed from the $Y_3N@C_{80}$ precursor after exposure to carbon vapor. Little fragmentation (C_2-elimination) is observed for $Y_3N@I_h\text{-}C_{80}$ after laser ablation at high energy without carbon vapor (i.e., low carbon density), whereas high energy laser ablation $Y_3N@I_h\text{-}C_{80}$ in the presence of graphite vapor results in extensive formation of larger $Y_3N@C_{2n}$ (C_{2n} = 82 – 100).

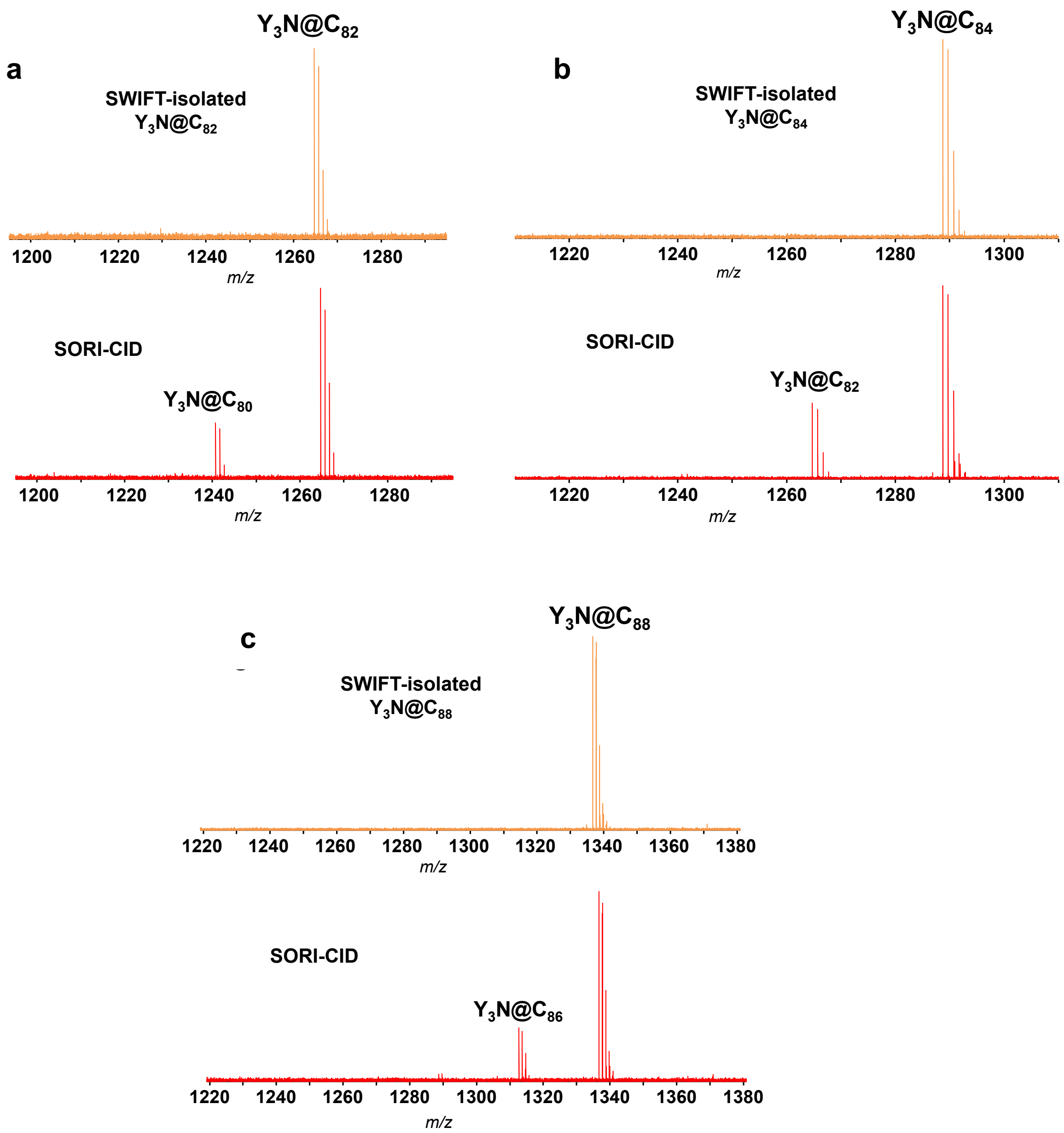

Supplementary Figure 15. SWIFT-isolation and SORI-CID spectra for (**a**) $Y_3N@C_{82}$, (**b**) $Y_3N@C_{84}$, and (**c**) $Y_3N@C_{88}$**,** which are bottom-up self-assembly products formed after exposure of $Y_3N@I_h$-C_{80} to carbon vapor. The dissociation patterns for all species confirm the metallic nitride clusterfullerene structures.

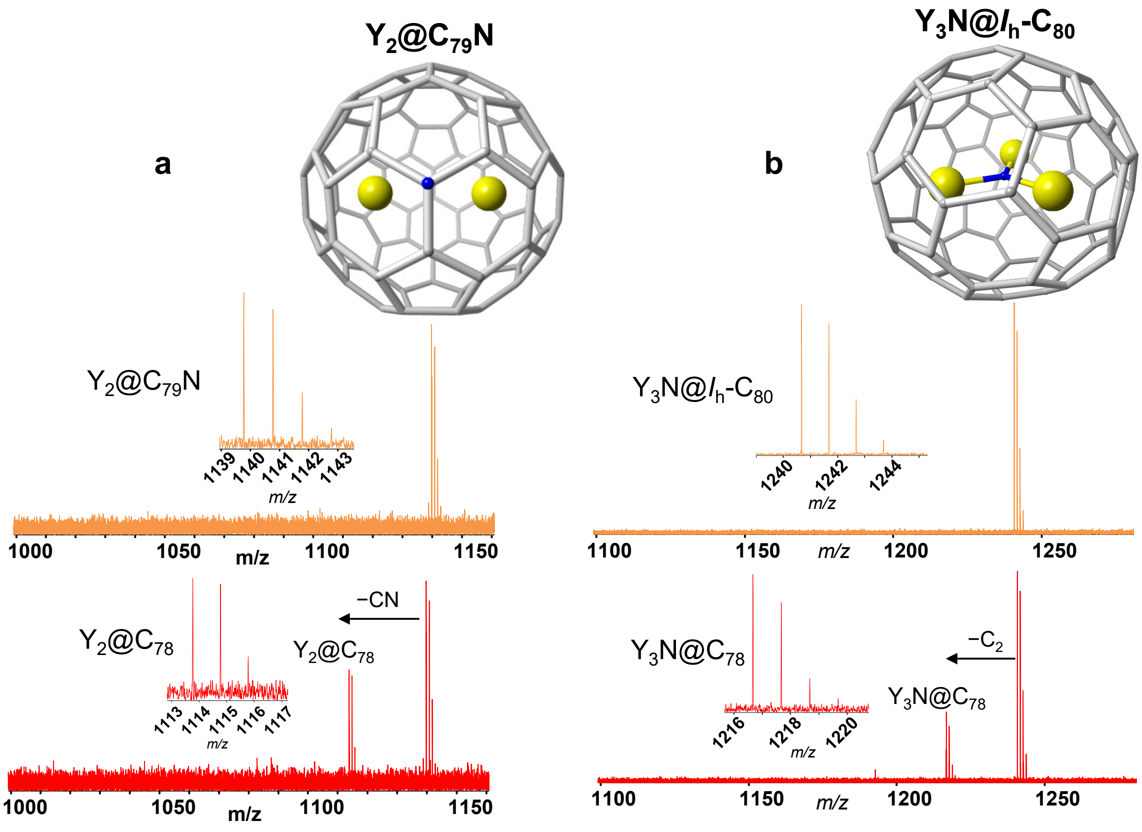

Supplementary Figure 16. (**a**) SWIFT-isolation and SORI-CID spectra for $Y_2@C_{79}N$, an endohedral heterofullerene, compared to (**b**) the nitride clusterfullerene, $Y_3N@I_h$-C_{80}. C_2-elimination with retention of the encapsulated metals and heteroatom, Y_3N, is observed for $Y_3N@I_h$-C_{80}, as expected. By contrast, CN-elimination is observed when nitrogen is bonded within the carbon network for a metallofullerene. Consequently, CN-loss occurs only for endohedral heterofullerenes, and therefore, SORI-CID investigations provide a structural diagnostic to determine the location of a nitrogen atom (i.e., endohedral or networked) in a metallofullerene.

Supplementary Table 1. Reaction Energies for the C_{68} to C_{72} Growth Steps[a)]

**Step**	**C_{2n}^{6-}**	**$Sc_3N@C_{2n}$**
C_{68}(6140) → C_{70}(hept)	-182.6	-167.9
C_{68}(6140) → C_{70}(7886)	-194.5	-179.3
C_{70}(hept) → C_{72}(10611)	-242.6	-210.7
C_{70}(hept) → C_{72}(10610)	-226.4	-207.0
C_{70}(7886) → C_{72}(10610)	-214.5	-195.7
C_{70}(7886) → C_{72}(hept1)	-206.4	-176.7
C_{72}(10611) → C_{72}(10610)	+3.7	-1.8

a) Energies in kcal·mol^{-1}; see Figs 5 and 6 for more details

Supplementary Table 2. Reaction energies (kcal·mol^{-1}) in hexaanion and endohedral form.

Reaction	C_{2n}^{6-}	$Sc_3N@C_{2n}$
**$C_{68} \rightarrow C_{70}$**		
6140 → hept	-182.6	-167.9
6140 → 7886	-194.5	-179.3
**$C_{70} \rightarrow C_{72}$**		
hept → 10611	-242.6	-210.7
hept → 10610	-226.4	-207.0
7886 → 10610	-214.5	-195.7
7886 → hept1	-206.4	-176.7
7854 → hept2	-194.0	-177.6
**$C_{72} \rightarrow C_{74}$**		
10611 → 13400	-200.4	-192.5
10611 → hept1	-180.4	-171.9
10610 → 13391	-207.6	-177.3
10610 → hept1	-196.5	-175.6
hept1 → 13400	-224.7	-215.1
hept2 → 13295	-231.5	-211.2
hept2 → 13290	-222.3	-205.4
10528 → 13333	-221.0	-188.5
**$C_{74} \rightarrow C_{76}$**		
hept1 → 17765	-223.2	-211.6
13400 → 17512	-200.8	-198.9
13400 → hept1	-194.3	-190.4
13295 → hept3	-193.0	-188.5
13290 → hept3	-202.2	-194.3
13333 → 17490	-213.1	-217.2
**$C_{76} \rightarrow C_{78}$**		
17765 → hept1	-205.0	-219.8
17512 → hept1	-200.9	-175.7
hept3 → 22010	-226.3	-223.2
17490 → 22010	-211.1	-202.7
**$C_{78} \rightarrow C_{80}$**		
hept1 → 31923	-257.1	-227.2
22010 → hept	-217.5	-213.2

Supplementary Table 3. Relative energies (kcal·mol^{-1}) for intermediates in hexaanion and endohedral form.

Cage	Isomer	C_{2n}^{6-}	$Sc_3N@C_{2n}$
C_{70}	7854	0.0	0.0
	7886	13.6	9.0
	7892	16.2	
	Hept	25.6	20.3
C_{72}	10611	0.0	1.7
	10528	17.5	0.0
	10610	16.1	5.4
	10468	24.9	
	Hept1	24.2	24.3
	Hept2	23.0	14.5
	10482	19.4	
C_{74}	14246	5.3	0.0
	13295	0.0	11.4
	14239	20.3	11.2
	13400	8.1	17.4
	13290	9.2	17.2
	13333	5.0	19.6
	13391	17.1	36.3
	Hept1	28.2	38.0
	Hept2	30.2	
	Hept3	39.9	
	13334	28.1	
C_{76}	17490	0.0	0.5
	19151	5.5	0.0
	17765	13.1	24.4
	17512	15.4	16.5
	17416	20.0	
	17405	28.5	
	17491	21.9	34.8
	Hept1	22.0	25.0
	Hept2	26.1	
	Hept3	15.2	20.9
	Hept4	28.5	
	Hept5	49.0	
	18638	30.0	
	18556	29.4	
C_{78}	24109	0.0	0.0
	22010	14.8	19.4
	24099	32.8	41.9
	Hept1	34.0	26.2
	Hept2	40.4	62.5
	Hept3	59.6	
	Hept4	54.9	
	Hept5	49.1	
	Hept6	48.5	
	Hept7	63.3	
	Hept8	61.2	
	21981	31.3	
	23470	66.2	
	21760	56.2	
	22963	43.7	
	22646	26.5	
	21827	45.5	
	21828	43.7	
	22410	49.2	
	21791	45.3	
C_{80}	31924	0.0	0.0
	31923	21.0	18.8
	30233	77.8	
	28325	63.8	
	28333	77.1	
	28133	70.4	
	Hept	41.4	25.9
	Hept1	61.1	
	31891	38.4	
	30291	79.9	

Supplementary Table 4. Relative energies for selected structures in growth paths to I_h or D_{5h}-C_{80} a)

**Cage**	**$Sc_3N@C_{2n}$**	**$Y_3N@C_{2n}$**	**Other Forms** b)
C_{70}(7886)	9.0		–
C_{70}(hept)	20.3		–
C_{72}(10611)	1.7		La_2, Pr_2
C_{72}(10528)	0.0		S_2Sc
C_{72}(10610)	5.4		–
C_{74}(13333)	19.6		–
C_{74}(13290)	17.2		–
C_{76} (17490)	0.5	0.0	La_2, Lu_2
C_{76} (hept3)	20.9	10.3	–
C_{78}(22010)	19.4	0.0	Gd_3N
C_s(hept)-C_{80}	41.4	25.9	$LaSc_2N$

a) Values represent relative energies (in $kcal \cdot mol^{-1}$) of the EMF with respect to the most stable isomer.
b) Known cages characterized by experimental techniques as other endofullerene forms.

Supplementary Table 5. Cartesian coordinates for optimized heptagon-containing structures (shown in Fig. 5).

Heptagon structures

C_{70}(hept)

C	-1.712114	-3.894675	-0.602436
C	-1.648522	-3.445455	-1.971077
C	-2.519054	-2.293917	-2.113265
C	-3.208087	-2.091675	-0.839548
C	-2.674091	-3.070191	0.088468
C	-0.353904	-3.976271	-0.092084
C	0.561798	-3.671893	-1.192916
C	-0.258018	-3.321117	-2.335932
C	0.146536	-2.266497	-3.185051
C	-0.793104	-1.213240	-3.542510
C	-2.095622	-1.167646	-2.905123
C	-2.603860	0.121453	-2.524549
C	-3.406507	0.300483	-1.331862
C	-3.602310	-0.774567	-0.387507
C	-3.532920	-0.460381	1.047389
C	-2.907702	-1.422367	1.928341
C	-2.426130	-2.686964	1.429077
C	-1.138961	-2.974659	2.044085
C	-0.060644	-3.527457	1.241174
C	1.266125	-3.034149	1.490244
C	2.225982	-2.851109	0.409491
C	1.834148	-3.022282	-0.968345
C	2.333563	-2.057416	-1.956014
C	1.460684	-1.684972	-3.047987
C	1.378444	-0.308498	-3.488029
C	-0.019938	0.000801	-3.721800
C	-0.535884	1.280709	-3.301675
C	-1.842009	1.337235	-2.733839
C	-2.164029	2.296090	-1.684874
C	-3.193165	1.655005	-0.884012
C	-3.322436	1.976581	0.471144
C	-3.450895	0.921869	1.457462
C	-2.663207	1.345748	2.614642
C	-1.816158	0.401914	3.297819
C	-1.988821	-0.984227	2.968802
C	-0.877146	-1.914996	2.997405
C	0.458537	-1.430650	3.239554
C	1.535109	-2.039847	2.503761
C	2.687254	-1.257095	2.076280
C	3.114148	-1.787940	0.804365
C	3.690639	-0.929478	-0.157807
C	3.292782	-1.055736	-1.545203
C	3.343183	0.272946	-2.130523
C	2.301365	0.696837	-3.047233
C	1.815349	2.050462	-2.965407
C	0.391131	2.296861	-2.907719
C	0.112510	3.260556	-1.827533
C	-1.162501	3.189373	-1.101053
C	-1.328629	3.537346	0.315197
C	-2.506351	3.035463	1.002057
C	-2.180611	2.685632	2.357203
C	-0.812600	3.038958	2.580973
C	-0.012949	2.170834	3.390923
C	-0.505149	0.838843	3.731932
C	0.626811	-0.069466	3.656467
C	1.803822	0.696308	3.250297
C	2.803264	0.146351	2.369371
C	3.491799	1.035530	1.406646
C	3.963412	0.453742	0.152824
C	3.840187	1.187587	-1.105940
C	3.412228	2.534537	-1.096316
C	2.421089	2.971244	-2.048225
C	1.398202	3.704820	-1.335282
C	1.149045	3.719021	1.235509
C	-0.279221	3.665515	1.380815
C	1.980149	2.900998	2.131022
C	1.387036	2.070413	3.113597
C	3.126348	2.435096	1.389092
C	1.891857	3.925253	0.027987
C	3.143549	3.190743	0.148898

C_{72}(hept1)

C	-1.696540	-3.981387	-0.520657
C	-1.669850	-3.578820	-1.903879
C	-2.582153	-2.472728	-2.074901
C	-3.240622	-2.231211	-0.789124
C	-2.659487	-3.155730	0.164173
C	-0.337409	-4.045664	-0.033434
C	0.554383	-3.742411	-1.157559
C	-0.286750	-3.425584	-2.292209
C	0.070191	-2.353048	-3.140944
C	-0.913426	-1.369834	-3.540218
C	-2.231470	-1.370337	-2.930348
C	-2.832038	-0.098895	-2.640772
C	-3.569570	0.117950	-1.404911
C	-3.637602	-0.901382	-0.389356
C	-3.477796	-0.499672	1.016525
C	-2.827302	-1.417379	1.921454
C	-2.374042	-2.711756	1.476854
C	-1.083833	-2.990826	2.086271
C	-0.023181	-3.573538	1.286726
C	1.316866	-3.107335	1.513068
C	2.245500	-2.930190	0.412654
C	1.814820	-3.071168	-0.955822
C	2.240458	-2.048813	-1.929397
C	1.339967	-1.685578	-2.978494
C	1.192156	-0.297929	-3.383332
C	-0.205107	-0.120499	-3.728364
C	-0.837267	1.134496	-3.491190
C	-2.186942	1.147799	-2.995014
C	-2.591582	2.164000	-2.033540
C	-3.418591	1.511708	-1.059062
C	-3.349036	1.904805	0.299218
C	-3.354099	0.903754	1.330170
C	-2.476862	1.382444	2.390215
C	-1.668089	0.460584	3.133586
C	-1.882824	-0.940783	2.916036
C	-0.790622	-1.891814	2.988158
C	0.556517	-1.434607	3.207878
C	1.623781	-2.099747	2.511512
C	2.815646	-1.376250	2.094808
C	3.184869	-1.905627	0.794712
C	3.720161	-1.045598	-0.192633
C	3.166762	-1.025256	-1.520725
C	3.082150	0.382569	-1.957257
C	2.014032	0.777570	-2.860546
C	1.424008	2.119713	-2.887868
C	0.001789	2.248452	-3.184475
C	-0.528564	3.403669	-2.470041
C	-1.732181	3.268304	-1.684070
C	-1.714874	3.735688	-0.277022
C	-2.486899	2.996410	0.689583
C	-1.965658	2.683867	2.011024
C	-0.637178	3.097583	2.385963
C	0.156161	2.187167	3.173597
C	-0.356785	0.884731	3.544112
C	0.746853	-0.047884	3.551522
C	1.939350	0.672550	3.160120
C	2.971948	0.038123	2.380146
C	3.662350	0.861860	1.422992
C	4.060177	0.291815	0.160270
C	3.734815	1.186487	-0.912828
C	3.330244	2.442264	-0.291811
C	2.549818	3.459062	-0.880638
C	1.799633	4.334420	0.003038
C	1.495778	3.928593	1.355194
C	0.047442	4.004853	1.518892
C	2.246112	2.871950	1.960302
C	1.580263	2.056601	2.946739
C	3.269375	2.232656	1.161884
C	-0.525414	4.394660	0.224881
C	0.567451	4.685008	-0.650330
C	0.593983	4.106194	-1.960703
C	1.795446	3.321905	-2.136522

C_{74}(hept1)

C	-1.706877	-3.862838	-0.366675
C	-1.732176	-3.283247	-1.711143
C	-2.657313	-2.207357	-2.051098
C	-3.663821	-1.877329	-1.065604
C	-2.693109	-3.495092	0.575796
C	-0.318035	-4.010903	0.052985
C	0.517637	-3.623217	-1.072926
C	-0.360877	-3.220656	-2.148253
C	0.063182	-2.225992	-3.056009
C	-0.854107	-1.206008	-3.494618
C	-2.183156	-1.128624	-2.947716
C	-2.742611	0.195361	-2.824330
C	-3.608855	0.506362	-1.708100
C	-3.987973	-0.500290	-0.800997
C	-3.928104	-0.279776	0.654661
C	-2.888347	-1.946818	2.319291
C	-2.298362	-3.207804	1.931625
C	-0.924436	-3.234639	2.331741
C	0.088875	-3.666649	1.403226
C	1.412321	-3.136502	1.575797
C	2.285927	-2.873795	0.448933
C	1.809125	-2.997650	-0.904698
C	2.271512	-2.019380	-1.900983
C	1.386504	-1.667879	-2.979344
C	1.313887	-0.302872	-3.481599
C	-0.069172	-0.015449	-3.782419
C	-0.603387	1.296265	-3.546200
C	-1.952291	1.390380	-3.085044
C	-2.315757	2.402116	-2.098278
C	-3.320285	1.823575	-1.237435
C	-3.235027	2.037056	0.175741
C	-3.408703	0.966870	1.131218
C	-2.466893	1.234111	2.267490
C	-1.718314	0.274717	3.077839
C	-1.897811	-1.185401	3.043072
C	-0.708528	-2.049231	3.126058
C	0.607278	-1.526225	3.296518
C	1.693460	-2.135444	2.584697
C	2.810116	-1.319307	2.129327
C	3.180724	-1.806854	0.828313
C	3.671176	-0.908435	-0.145944
C	3.231524	-1.015638	-1.508335
C	3.206158	0.334935	-2.055288
C	2.199343	0.720721	-3.001543
C	1.711346	2.074001	-2.953312
C	0.307668	2.343528	-3.184621
C	-0.091275	3.411452	-2.286203
C	-1.365116	3.356846	-1.607780
C	-1.410674	3.727019	-0.180476
C	-2.298370	2.992271	0.682874
C	-1.879518	2.537795	1.997327
C	-0.633649	2.995244	2.544759
C	0.083292	2.063639	3.368180
C	-0.432187	0.728131	3.581318
C	0.718765	-0.157163	3.650687
C	1.910995	0.607313	3.345292
C	2.921944	0.079155	2.460534
C	3.534418	0.999073	1.480253
C	3.885066	0.480182	0.183375
C	3.649221	1.254348	-1.028951
C	3.161819	2.604385	-0.962544
C	2.207481	3.023267	-1.975009
C	1.092106	3.877967	-1.611890
C	1.556034	4.014556	1.914960
C	0.111047	3.986551	1.816534
C	2.253400	2.906980	2.500457
C	1.530956	1.990904	3.297090
C	3.230270	2.407173	1.548517
C	2.099912	4.238038	0.599003
C	3.094059	3.218894	0.334628
C	1.002292	4.422450	-0.307925
C	-0.239364	4.296638	0.431313
C	-3.834675	-1.569704	1.278402
C	-3.709441	-2.557603	0.212189

C_{76}(hept1)

C	-1.684412	-3.865284	-0.593733
C	-1.666589	-3.450884	-1.965297
C	-2.617115	-2.367719	-2.134876
C	-3.261862	-2.144252	-0.838687
C	-2.674878	-3.082843	0.097073
C	-0.322482	-3.866693	-0.103950
C	0.574782	-3.469423	-1.191670
C	-0.293589	-3.240922	-2.334812
C	0.009930	-2.215764	-3.235487
C	-1.012410	-1.271641	-3.661703
C	-2.295982	-1.263326	-2.989842
C	-2.858108	0.019933	-2.662127
C	-3.556064	0.225474	-1.398247
C	-3.647527	-0.820644	-0.410111
C	-3.501081	-0.457328	1.013629
C	-2.899884	-1.413594	1.913541
C	-2.427293	-2.685342	1.429902
C	-1.145047	-2.970358	2.050758
C	-0.051553	-3.443471	1.221950
C	1.259449	-2.923294	1.464183
C	2.226621	-2.610966	0.418378
C	1.817137	-2.732381	-0.988070
C	2.242716	-1.805421	-2.099866
C	1.280296	-1.557389	-3.161503
C	1.087368	-0.249680	-3.757495
C	-0.328749	-0.039613	-3.963486
C	-0.898729	1.243603	-3.646275
C	-2.184043	1.250647	-3.002420
C	-2.489183	2.238503	-1.976011
C	-3.346536	1.598607	-1.015980
C	-3.257463	1.949508	0.351319
C	-3.341745	0.932223	1.362226
C	-2.492882	1.361267	2.469015
C	-1.765565	0.401864	3.241680
C	-1.993139	-0.984231	2.965612
C	-0.905482	-1.940613	3.033092
C	0.426282	-1.512732	3.353752
C	1.515724	-2.093646	2.616425
C	2.744438	-1.387928	2.394615
C	3.225185	-1.729730	1.060721
C	4.185974	-0.881681	0.455700
C	3.276054	-0.786530	-1.996302
C	3.118250	0.491940	-2.693746
C	1.956445	0.819977	-3.441449
C	1.427703	2.151247	-3.345733
C	-0.007555	2.336722	-3.373275
C	-0.338139	3.342821	-2.374056
C	-1.526616	3.232932	-1.572981
C	-1.459804	3.624792	-0.153950
C	-2.338863	2.974532	0.779895
C	-1.894659	2.631737	2.121781
C	-0.578739	2.986132	2.577869
C	0.094291	2.076814	3.467242
C	-0.480741	0.786037	3.761986
C	0.601219	-0.171300	3.843155
C	1.839791	0.534445	3.644160
C	2.956550	-0.079789	2.978308
C	3.889106	0.775640	2.327255
C	4.651106	0.274426	1.215095
C	3.846657	1.522671	-1.968399
C	3.180811	2.759518	-1.615549
C	1.995277	3.076075	-2.377355
C	0.902408	3.806680	-1.803682
C	1.671406	3.691916	1.706622
C	0.227973	3.810356	1.723910
C	2.341339	2.731602	2.541534
C	1.536106	1.934531	3.431482
C	3.566074	2.170256	2.060716
C	2.127034	3.924791	0.341822
C	3.268478	3.236020	-0.214020
C	0.959616	4.215751	-0.449494
C	-0.215748	4.126855	0.372829
C	4.084190	2.491716	0.725019
C	4.838724	1.358450	0.287857
C	4.656624	0.841416	-1.026871
C	4.272974	-0.555090	-0.961682

C_{78}(hept1)

C	-1.676758	-3.571253	-0.271080
C	-1.688588	-3.134241	-1.659308
C	-2.612358	-2.130712	-2.121499
C	-3.668197	-1.756495	-1.211970
C	-2.619703	-3.042646	0.672796
C	-0.302986	-3.824863	0.104940
C	0.539849	-3.552977	-1.050929
C	-0.328885	-3.170688	-2.130012
C	0.113494	-2.241922	-3.099165
C	-0.792178	-1.244035	-3.603668
C	-2.147111	-1.146813	-3.115300
C	-2.739982	0.168176	-3.116639
C	-3.770939	0.522406	-2.172152
C	-4.248830	-0.442655	-1.248170
C	-4.585389	-0.044310	0.104485
C	-2.655588	-1.743187	2.816790
C	-2.171665	-2.849915	2.029824
C	-0.797015	-3.099244	2.402497
C	0.165548	-3.542681	1.430530
C	1.526686	-3.119427	1.584596
C	2.371434	-2.869042	0.425317
C	1.853745	-2.976660	-0.912619
C	2.319882	-2.019310	-1.931142
C	1.437745	-1.685346	-3.018407
C	1.373332	-0.332658	-3.555709
C	-0.000532	-0.063197	-3.903241
C	-0.555227	1.245804	-3.699209
C	-1.924187	1.350183	-3.341244
C	-2.379320	2.382278	-2.407326
C	-3.537013	1.886511	-1.741131
C	-3.759821	2.253454	-0.388743
C	-4.393258	1.305714	0.498790
C	-2.349544	1.024165	3.387192
C	-1.381511	0.113158	3.917831
C	-1.572449	-1.290702	3.662456
C	-0.429291	-2.143552	3.421338
C	0.912051	-1.629761	3.502691
C	1.901069	-2.174563	2.615552
C	2.993119	-1.358465	2.112588
C	3.292616	-1.821214	0.783251
C	3.751037	-0.911514	-0.198320
C	3.282841	-1.017286	-1.553643
C	3.238639	0.333080	-2.102181
C	2.234295	0.705876	-3.053787
C	1.715937	2.049883	-2.999206
C	0.318597	2.293452	-3.269752
C	-0.142140	3.299638	-2.330740
C	-1.447810	3.246349	-1.733275
C	-1.613840	3.547414	-0.274673
C	-2.795932	3.038863	0.364333
C	-1.956597	2.297334	2.782610
C	-0.587634	2.757535	2.872136
C	0.310537	1.937782	3.674514
C	-0.058409	0.598935	4.098794
C	1.087896	-0.274576	3.925371
C	2.194832	0.528052	3.447168
C	3.120019	0.038588	2.458533
C	3.640806	0.979401	1.448298
C	3.951832	0.476887	0.133377
C	3.661343	1.252401	-1.065067
C	3.121838	2.578655	-0.978528
C	2.151487	2.979520	-1.975969
C	0.986614	3.755624	-1.590869
C	1.440930	3.806433	1.924540
C	0.003640	3.654422	1.885655
C	2.291213	2.802066	2.508421
C	1.732003	1.891510	3.404440
C	3.259866	2.369029	1.525928
C	1.942252	4.087072	0.617038
C	3.019318	3.167249	0.324675
C	0.815007	4.187844	-0.254348
C	-0.419345	3.964743	0.476181
C	-4.215822	-1.126851	1.005207
C	-3.659449	-2.190205	0.185423
C	-3.646105	-0.831902	2.305526
C	-3.532761	0.543099	2.711810
C	-3.936098	1.593089	1.820199
C	-2.949632	2.638290	1.790829

C_{78}(hept2)

C	-1.823541	-3.652355	-0.589627
C	-1.600256	-3.340907	-1.993494
C	-2.424038	-2.408356	-2.669048
C	-3.721032	-2.054061	-0.563361
C	-2.841688	-2.939669	0.160812
C	-0.516946	-3.905442	-0.004782
C	0.489521	-3.754181	-1.038620
C	-0.190165	-3.344667	-2.255468
C	0.413471	-2.390726	-3.148808
C	-0.411663	-1.292180	-3.693709
C	-1.819927	-1.340127	-3.438568
C	-2.580041	-0.127329	-3.147880
C	-3.932416	0.223072	-1.108026
C	-4.044590	-0.745460	-0.017871
C	-3.673872	-0.378404	1.314361
C	-2.998672	-1.358645	2.120806
C	-2.539773	-2.599500	1.526210
C	-1.266076	-2.931140	2.131741
C	-0.215230	-3.539283	1.353301
C	1.137262	-3.173701	1.670567
C	2.140100	-3.031170	0.634002
C	1.802242	-3.263552	-0.726807
C	2.488167	-2.490252	-1.713131
C	1.808767	-2.154147	-2.950243
C	1.619559	0.122284	-3.922135
C	0.180703	0.022524	-3.920380
C	-0.576355	1.236589	-3.536831
C	-1.924898	1.122295	-2.972867
C	-2.358414	2.021099	-1.878823
C	-3.334307	1.506926	-0.903859
C	-3.245658	1.938355	0.459410
C	-3.403223	1.004804	1.564610
C	-2.500279	1.417914	2.617611
C	-1.761796	0.438179	3.376673
C	-2.031891	-0.949509	3.118679
C	-0.963963	-1.929170	3.128308
C	0.391460	-1.538038	3.412128
C	1.437478	-2.205127	2.692760
C	2.637076	-1.494911	2.297203
C	3.047990	-1.960386	0.990562
C	3.686397	-1.097825	0.011438
C	3.425028	-1.414017	-1.389772
C	3.411748	0.933699	-2.627442
C	2.304987	1.308802	-3.522050
C	1.588440	2.509082	-3.323255
C	0.131290	2.438347	-3.280710
C	-0.322433	3.388705	-2.284821
C	-1.482932	3.117898	-1.479461
C	-1.459017	3.584205	-0.087116
C	-2.311887	2.942027	0.859258
C	-1.870943	2.650720	2.210601
C	-0.551820	3.008200	2.638363
C	0.147392	2.079848	3.489870
C	-0.437772	0.795275	3.810312
C	0.628117	-0.184364	3.828783
C	1.865446	0.496996	3.509058
C	2.856316	-0.144571	2.686865
C	3.643791	0.671463	1.814006
C	4.053455	0.243224	0.474523
C	3.821556	1.838402	-1.636813
C	3.187695	3.159220	-1.561977
C	2.040207	3.468141	-2.375141
C	0.854908	4.031365	-1.738968
C	1.669738	3.804580	1.753454
C	0.214663	3.857683	1.783941
C	2.351043	2.731512	2.460754
C	1.573569	1.905823	3.340589
C	3.481733	2.111922	1.819270
C	2.091283	4.191673	0.421154
C	3.221938	3.575404	-0.188367
C	0.891698	4.403203	-0.379099
C	-0.262445	4.187356	0.446751
C	3.949344	2.575883	0.555164
C	4.294558	1.466278	-0.290716
C	-3.687741	-0.501378	-2.306975
C	-3.584872	-1.899547	-1.994176
C	2.413221	-0.982760	-3.447662
C	3.445259	-0.522407	-2.553625

C_{80}(hept)

C	-1.591915	-2.402807	-3.174317
C	-0.213107	-2.823096	-3.330981
C	0.514911	-1.685145	-3.859600
C	-0.414830	-0.580404	-4.020941
C	-1.706254	-1.008861	-3.524724
C	-2.389636	-2.913466	-2.132163
C	-0.443398	-4.219162	-1.293009
C	0.394743	-3.683153	-2.332502
C	1.770952	-3.446340	-2.005005
C	2.518361	-2.361405	-2.600282
C	1.879529	-1.430434	-3.494610
C	2.330737	-0.065321	-3.465227
C	1.412489	1.031129	-3.678866
C	0.013264	0.778282	-3.883141
C	-0.925536	1.713584	-3.343185
C	-2.195689	1.266688	-2.795639
C	-2.562556	-0.117204	-2.783910
C	-3.332866	-0.627262	-1.640759
C	-3.228134	-2.044039	-1.354865
C	-3.192831	-2.553683	0.012828
C	-1.109508	-3.852186	0.956577
C	-0.000872	-4.254758	0.087459
C	1.368047	-3.989290	0.411362
C	2.268588	-3.649952	-0.660194
C	3.349136	-2.713506	-0.439265
C	3.502569	-1.915056	-1.638181
C	3.894953	-0.535351	-1.559099
C	3.334463	0.374875	-2.520258
C	3.048051	1.748253	-2.166685
C	1.854006	2.153109	-2.880726
C	0.911909	3.063731	-2.293349
C	-0.486000	2.858174	-2.579225
C	-1.495421	3.140214	-1.585363
C	-2.547252	2.145357	-1.714733
C	-3.278799	1.674211	-0.577943
C	-3.687970	0.262222	-0.544946
C	-3.911836	-0.329015	0.758677
C	-3.530645	-1.706668	1.057041
C	-3.033462	-1.788729	2.433090
C	-1.736719	-2.282971	2.891657
C	-0.827293	-3.220821	2.241482
C	0.570055	-3.117064	2.610172
C	1.644582	-3.480676	1.711193
C	2.768930	-2.599035	1.946132
C	3.592105	-2.151181	0.859631
C	4.109865	-0.810124	0.927051
C	4.254588	-0.010073	-0.271853
C	3.981620	1.368107	0.077138
C	3.326948	2.249282	-0.849763
C	2.448735	3.252268	-0.314849
C	1.254950	3.655652	-1.030720
C	0.238114	3.975255	-0.053566
C	-1.145399	3.680047	-0.307203
C	-1.977936	3.334049	0.804090
C	-3.062332	2.385007	0.657058
C	-3.247309	1.742251	1.934373
C	-3.719213	0.415841	1.970958
C	-3.216252	-0.477029	2.982624
C	-2.086117	-0.106799	3.775307
C	-1.199810	-1.254120	3.785819
C	0.204311	-1.076834	4.001396
C	1.077828	-2.061775	3.461366
C	2.417516	-1.718874	3.031266
C	2.877254	-0.360234	3.067595
C	3.758104	0.072330	2.014829
C	3.683000	1.421612	1.492417
C	2.736850	2.366267	2.016122
C	2.161724	3.315588	1.101683
C	0.798642	3.773979	1.264842
C	-0.017006	3.318879	2.358785
C	-1.428887	3.182585	2.140399
C	-2.215476	2.190651	2.849135
C	-1.569795	1.235637	3.730457
C	-0.166645	1.404362	3.973354
C	0.704942	0.263911	4.162796
C	2.018051	0.620346	3.669881
C	1.950678	1.971948	3.152182
C	0.596875	2.450606	3.330376
C	-1.840710	-3.919679	-1.260808
C	-2.272785	-3.692162	0.086670
